# Regioselectively α- and β-alkynylated BODIPY dyes via gold(I)-catalyzed direct C–H functionalization and their photophysical properties

**DOI:** 10.3762/bjoc.16.53

**Published:** 2020-04-01

**Authors:** Takahide Shimada, Shigeki Mori, Masatoshi Ishida, Hiroyuki Furuta

**Affiliations:** 1 Department of Chemistry and Biochemistry, Graduate School of Engineering, and Center for Molecular Systems, Kyushu University, Fukuoka 819-0395, Japan; 2 Advanced Research Support Center (ADRES), Ehime University, Matsuyama 790-8577, Japan

**Keywords:** alkynylation, BODIPY, direct C–H functionalization, gold(I)

## Abstract

A series of α- and β-ethynyl-substituted BODIPY derivatives (**3a**, **4a**, **5a, 5b**, **6a, 6b**) were synthesized by gold(I)-catalyzed direct C–H alkynylation reactions of dipyrromethane and BODIPY, respectively, with ethynylbenziodoxolone (EBX) in a regioselective manner. Depending on the position of the ethynyl substituent in the BODIPY skeleton, the photophysical properties of the resulting α- and β-substituted BODIPYs are notably altered. The lowest S_0_–S_1_ transition absorbance and fluorescence bands are both bathochromically shifted as the number of substituents increases, while the emission quantum yields of the β-ethynylated derivatives are significantly lower than those of α-ethynylated ones. The current method should be useful for fine-tuning of the photophysical properties of BODIPY dyes as well as for constructing BODIPY-based building cores for functional π-materials.

## Introduction

Boron-dipyrromethene (BODIPY, **1**) and its derivatives are representative families of fluorophores that have been widely used in applications for bioimaging [1–6], photodynamic therapy [7–12], photocatalysis [13–16], optics [17–20], and so forth. The structure of BODIPY derivatives is composed of a dipyrromethene (an oxidized form of dipyrromethane **2**) and a coordinated difluoroboron moiety [21]. The rigid π-conjugated scaffold of BODIPYs demonstrates fascinating optical features such as intense and narrow S_0_–S_1_ absorption and emission bands in the visible-to-near-infrared region, a high fluorescence quantum yield, and good photostability. For the applications mentioned above, various BODIPY dyes functionalized at the *meso*-, α-, and β-pyrrolic positions have been extensively developed to tune the optoelectronic properties [21]. Therefore, the development of more efficient and shorter step synthetic methods for the BODIPY derivatives, such as direct C–H functionalizations (e.g., arylation [22–28], annulation [29], olefination [30], styrylation [31], and borylation [32]), has been in great demand recently rather than the conventional multistep synthesis with nucleophilic substitution/cross-coupling via halogenation of BODIPYs [33] or from the activated precursors [34] via unstable pyrrolic intermediates. In particular, halogenation (e.g., bromination) of BODIPY derivatives often affords a mixture of multiply halogenated products, which is tedious to separate by column chromatography.

**Figure 1:**
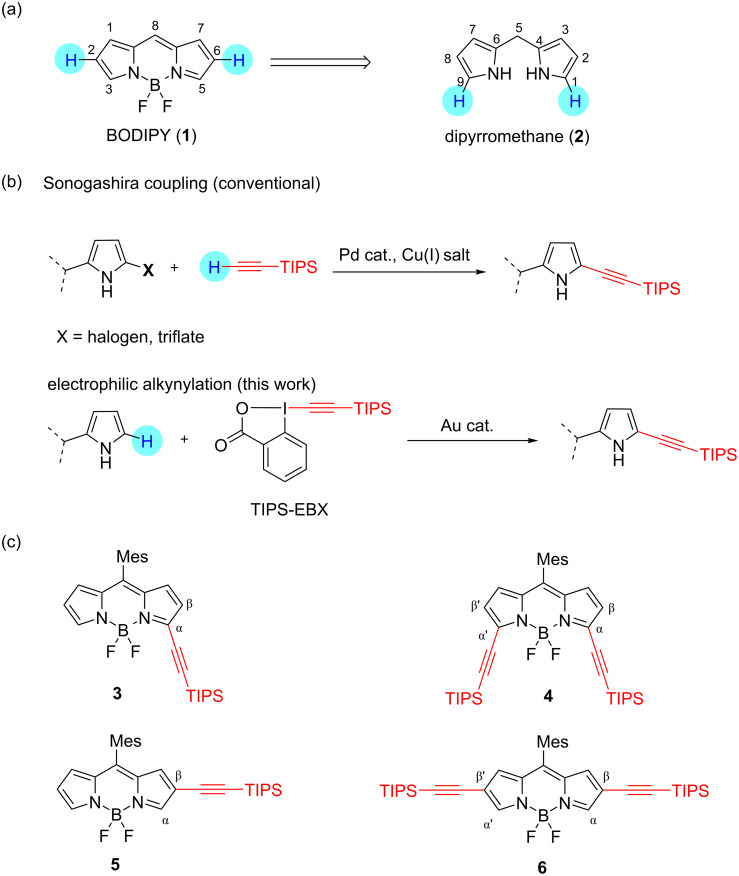
(a) Chemical structures of BODIPY (**1**) and dipyrromethane (**2**). (b) C–C bond forming alkynylations of pyrrole and its derivatives by Sonogashira coupling and electrophilic alkynylation. (c) Peripheral alkynylated BODIPY derivatives (**3–6**) prepared in this work. TIPS: triisopropylsilyl group; Mes: 2,4,6-trimethylphenyl group.

The introduction of one or more alkynyl groups into the BODIPY skeleton indeed produces useful building blocks for functional π-materials. For example, ethynyl-tethered BODIPY derivatives serve as a substrate in the copper-catalyzed azide–alkyne cycloaddition (CuAAC) reaction, which is known as “click” reaction, allowing for a biological tissue labelling [35–36]. In addition, ethynyl-substituted BODIPYs yield unique π-conjugated BODIPY-based macrocycles by Glaser-coupling reactions [37]. Conventionally, an alkynylation of the BODIPY core has been achieved by palladium-catalyzed Sonogashira cross-coupling with halogenated BODIPYs (Figure 1b) [35,37]. However, due to the coexistence of multiple C–H bonds, a regioselective direct C–H alkynylation of the BODIPY core has not yet been achieved.

Inspired by the works of Waser and co-workers showing the gold(I)-catalyzed C–H electrophilic alkynylation of various heterocycles (e.g., pyrroles, indoles, etc.) with ethynylbenziodoxolone (EBX) as an activated ethynyl synthon [38–42], we investigated the synthesis of ethynyl-substituted BODIPY derivatives **3–6** by gold(I)-catalyzed direct C–H functionalization (Figure 1c). By taking advantage of the reactivity of β-(2 and 6)-positions of BODIPY (**1**), which are susceptible to electrophilic reactions, β,β'-diethynyl-substituted BODIPYs **5** and **6** were prepared regioselectively, through the C–H alkynylation of **1** without any directing groups. In addition, the corresponding dipyrromethane **2**, which is a precursor of BODIPY, was first transformed into the alkynylated form under catalytic conditions, and subsequent oxidation followed by boron complexation to afford α-monoethynyl and α,α'-diethynyl-substituted BODIPYs **3** and **4**, respectively. The resulting ethynylated BODIPY isomers demonstrated site-dependent photophysical properties.

## Results and Discussion

### Synthesis and characterization

**Scheme 1:**
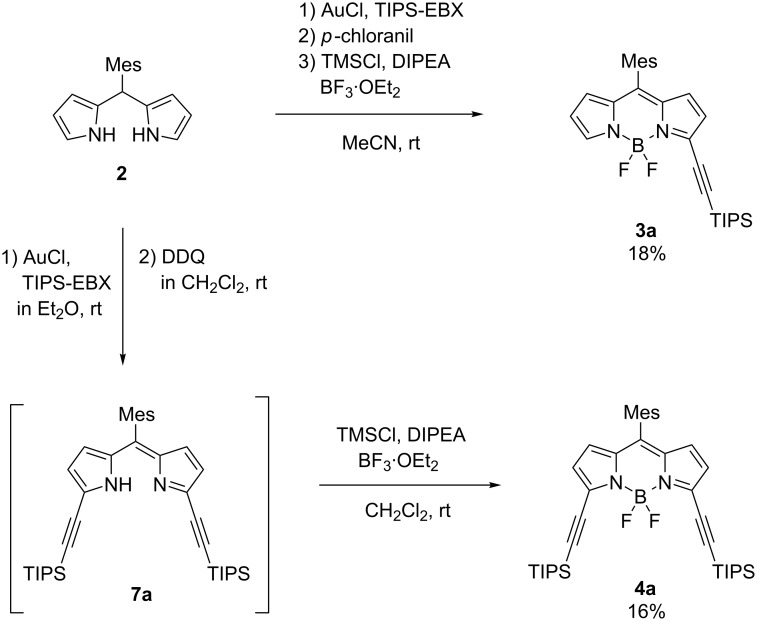
Synthesis of α-ethynyl-substituted BODIPY derivatives **3a** and **4a**.

To prepare the α,α'-diethynyl BODIPY **4a**, 5-mesityldipyrromethane **2** was used as the substrate for the gold(I) catalyzed reaction (Scheme 1). Mixing five mol % of gold(I) chloride and two equivalents of TIPS-EBX with a diethyl ether solution of **2** under ambient conditions yielded a mixture of ethynyl-substituted dipyrromethanes as judged by mass spectrometry. The product mixture was treated with 2,3-dichloro-5,6-dicyano-1,4-benzoquinone (DDQ) to give the α,α'-diethynyl-substituted dipyrrin **7a**. Subsequent boron complexation in the presence of trimethylsilyl chloride (TMSCl) as a fluoride scavenger afforded **4a** in 16% yield over three steps. Separately, α-ethynyl-monosubstituted dipyrrin **3a**, was obtained via a one-pot reaction in 18% yield (in three steps from **2**).

On the other hand, the β,β'-diethynyl-substituted BODIPY derivatives **5a** and **6a**, were synthesized by a similar gold-catalyzed reaction of unsubstituted BODIPY **1a** in 38% and 2% yields, respectively, in the presence of zinc(II) triflate as an activator of TIPS-EBX at 100 °C [41]. In contrast to the reaction of dipyrromethane **2**, the double C–H activation at the 2,6-positions of **1a** required harsh conditions (Table S1, Supporting Information File 1). The representative procedure of the reaction with EBX/gold(I) catalyst at room temperature gave only traces of **5a** with a large amount of unreacted starting material remaining. The addition of acid in expectation of the activation of TIPS-EBX was effective in promoting the gold–alkyne interactions [40–41]. After the investigation of various reaction conditions, such as gold catalysts (e.g., gold(I) cyanide), solvents (e.g., CH_2_Cl_2_, THF, MeCN, DMF), additives (e.g., TFA, Sc(OTf)_3_), and temperature, the reaction conditions as mentioned earlier were chosen for the synthesis of **5a** and **6a** (Table S1, Supporting Information File 1).

**Scheme 2:**
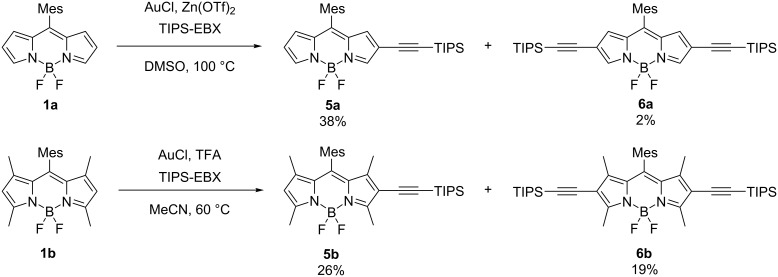
Synthesis of β-ethynyl-substituted BODIPY derivatives **5a** and **5b** and β,β'-diethynyl-substituted compounds **6a** and **6b**.

Because of the electron-deficient nature of BODIPY, the reactivity of the 2,6-positions is intrinsically low toward electrophiles, which hampers the β-selective functionalization. We, therefore, tested the corresponding reaction using tetramethyl-substituted BODIPY **1b** that is expected to have an enhanced electron density of the BODIPY core compared with **1a** (Scheme 2). Under milder conditions, the yield of the β,β'-diethynylated product **6b** was indeed improved to be 19% (from 2% of **6a**). These results indicate that electron-rich substrates facilitate the direct electrophilic alkynylation of the BODIPY core.

**Figure 2:**
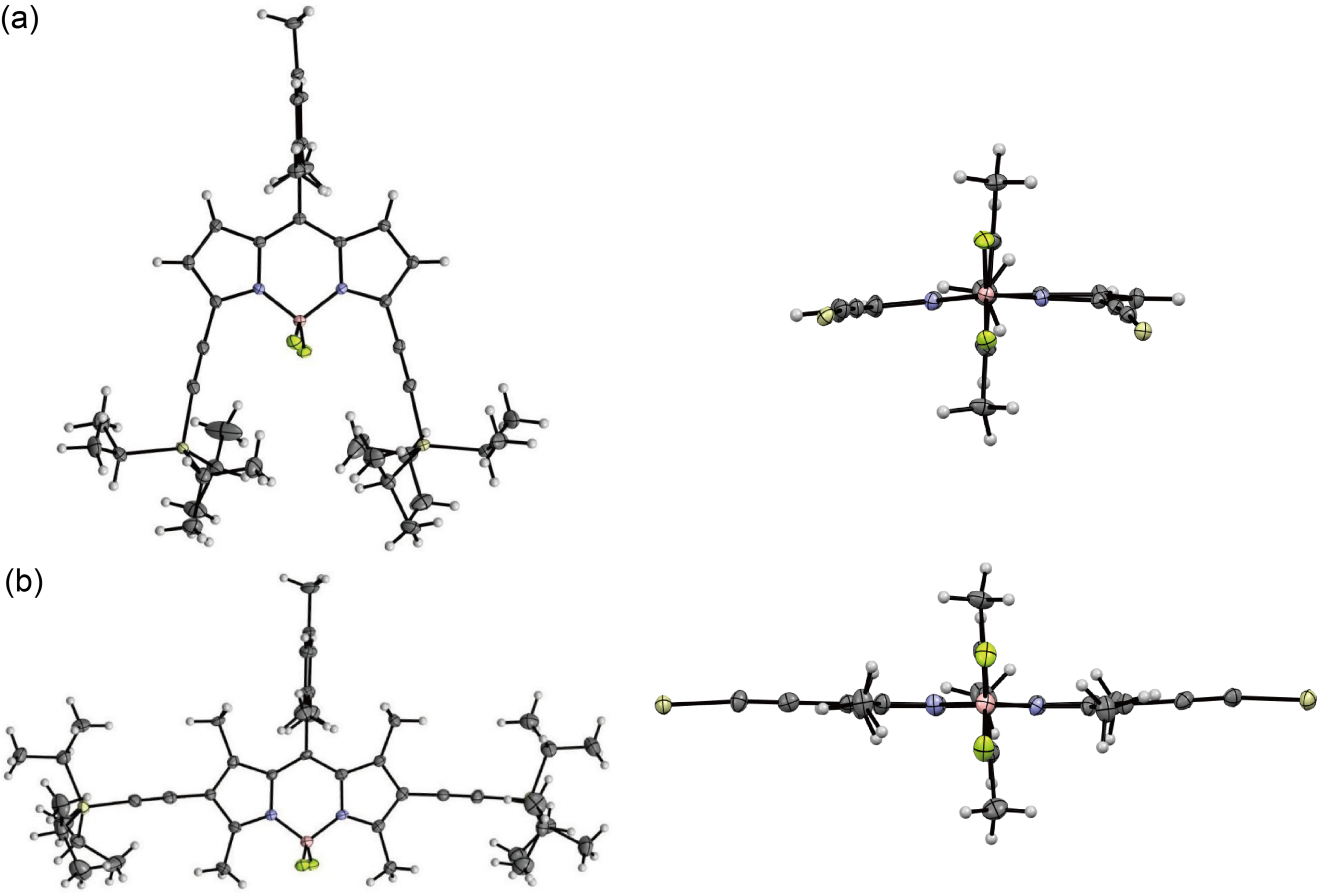
Top and front views of the crystal structures of (a) **4a** and (b) **6b** with 50% thermal ellipsoid probabilities. The isopropyl groups on the silicon atom are omitted in the front view for clarity.

The structures of the series of TIPS-ethynyl-substituted BODIPY derivatives **3–6** were characterized by ^1^H and ^19^F NMR spectroscopy, high-resolution mass spectrometry, and X-ray crystallographic analysis. The solid-state structures of the diethynyl-substituted BODIPYs were unambiguously elucidated by X-ray diffraction analysis (**3a** and **6b**: Figure 2 and Table S2 in Supporting Information File 1). The BODIPY cores of **3a** and **6b** are almost planar with smaller mean-plane deviations (defined by the 12 atoms of the tricyclic ring system) of 0.075 and 0.025 Å, respectively. The *meso-*mesityl groups are oriented perpendicularly to the BODIPY plane for both derivatives, indicating the rigid interlocked structures of **3a** and **6b** by the bulky *o*-methyl groups. In particular, the β-methyl substituents of **6b** are sterically hindered by the neighbouring *meso*-aryl ring. The regioselective 2,6-diethynylation of **6a** through the above alkynylation was also confirmed by its preliminary X-ray structure (Figure S20b, Supporting Information File 1).

**Figure 3:**
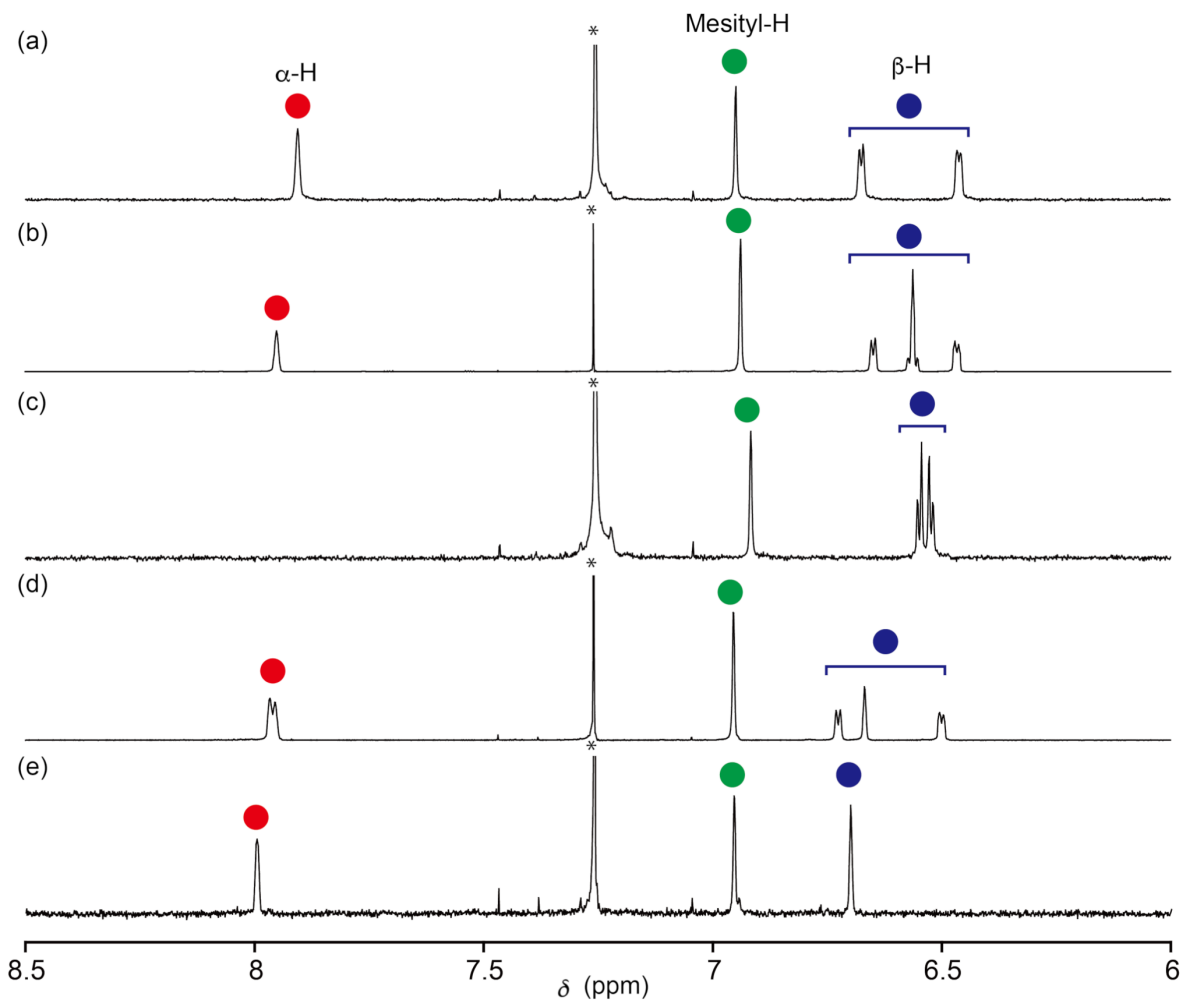
Partial ^1^H NMR spectra of (a) **1a**, (b) **3a**, (c) **4a**, (d) **5a**, and (e) **6a** recorded in CDCl_3_ at 298 K. Asterisks indicate the residual solvent peak.

The ^1^H NMR spectra of the BODIPY derivatives in CDCl_3_ reflect the characteristic structural features. The proton signals assignable to the α-pyrrolic CHs appeared in the lowest field region (δ ca. 8.00 ppm), and the β-CH resonances are in the range of 6.5 to 6.7 ppm (Figure 3). The disappearance of the α-pyrrole proton signals of **4a** is notable. In the case of **3a**, only a singlet signal appeared at 7.95 ppm for the α-pyrrole CH. In contrast, **6a** revealed two singlet signals at 7.99 and 6.70 ppm, assignable to the α- and β-pyrrole CHs, respectively. The ^1^H NMR spectrum of the mono-substituted compound **5a** represents an unsymmetrical resonance pattern similar to that of **3a** with two α-CH signals at 7.97 and 7.96 ppm. The proposed structures of all BODIPY compounds were elucidated based on the comparative NMR analysis and mass spectrometry (see Supporting Information File 1).

### Optical properties

**Figure 4:**
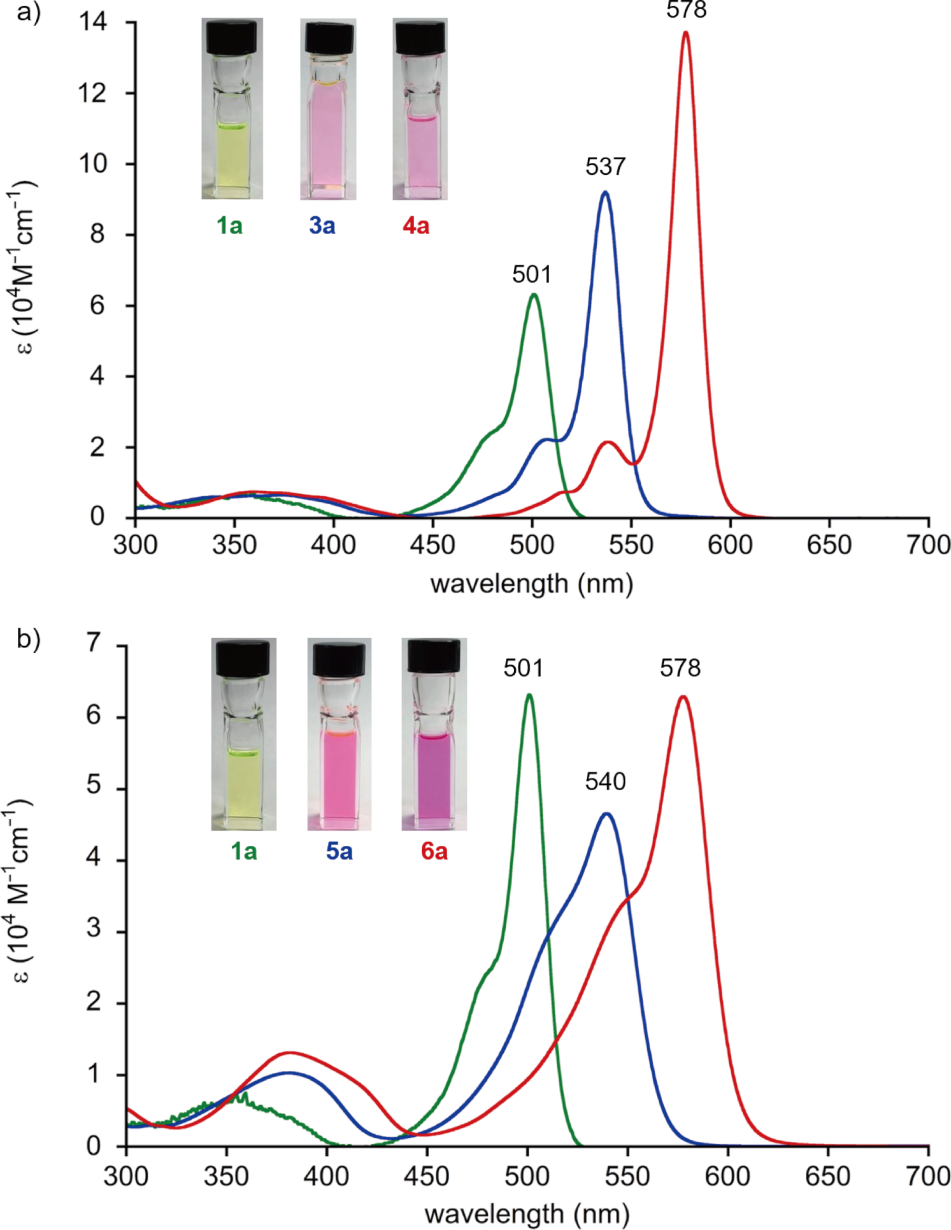
UV–vis absorption spectra of the BODIPY derivatives, (a) **1a** (green), **3a** (blue), **4a** (red), and (b) **1a** (green), **5a** (blue), **6a** (red) in CH_2_Cl_2_. Insets show the photo images of the solutions taken under ambient light.

**Figure 5:**
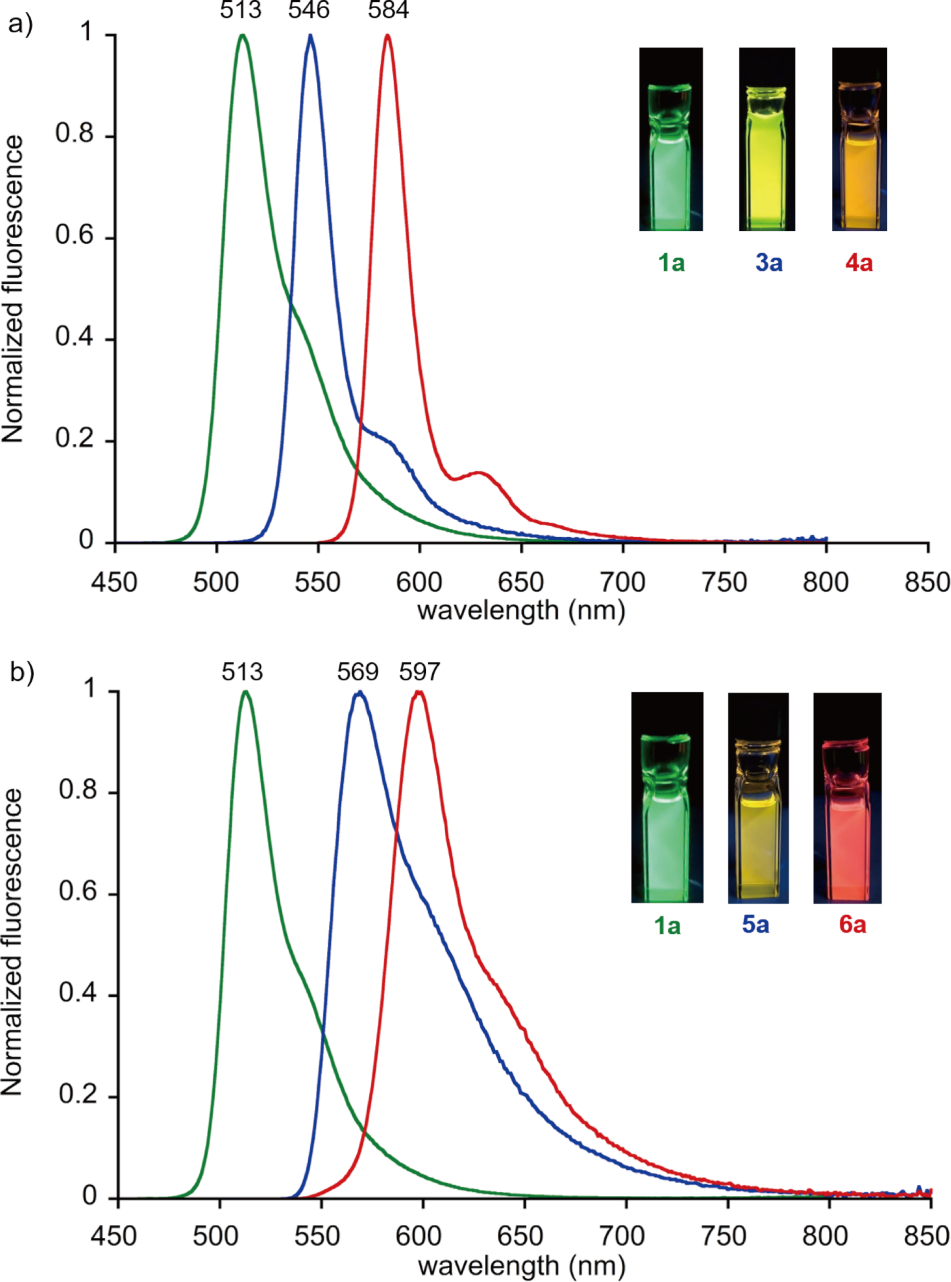
Fluorescence spectra of BODIPY derivatives. (a) **1a** (green), **3a** (blue), **4a** (red) and (b) **1a** (green), **5a** (blue), and **6a** (red) in CH_2_Cl_2_. Excitation was performed at 365 nm. The insets show the photo images of the solution under a UV lamp.

The α- and β-ethynyl-substituted BODIPYs exhibit large bathochromic shifts in the absorption and fluorescence spectra relative to the unsubstituted **1a** with extended π-conjugation (Figure 4 and Figure 5). The molar absorption coefficients (ε) of the S_0_–S_1_ bands of the α-ethynyl-substituted BODIPYs are substantially enhanced as the number of substituted groups increases (Figure 4a). Along with the absorption spectral profiles of **3a** and **4a**, sharp emission bands emerge with mirror structures (Figure 5a). The fluorescence quantum yields of **3a** and **4a** remain high (Φ_f_ = 0.95 and 0.56, respectively) and are comparable to that of **1a** (Table 1). The smaller Stokes shift values of ≈178 cm^−1^ and longer emission lifetimes of ≈7.83 ns indicate the rigid scaffolds of α,α-disubstituted **4a** in the excited state.

**Table 1:** Spectral properties of BODIPYs in CH_2_Cl_2_.

	λ_max_ (nm)	ε_max_^a^ (M^−1^cm^−1^)	λ_em_^b^ (nm)	Stokes shift (cm^−1^)	Φ_f_^c^	τ_f_^d^ (ns)

**1a**	501	6.3 × 10^4^	513	467	0.86	6.60
**3a**	537	9.2 × 10^4^	546	307	0.95	5.81
**4a**	578	1.4 × 10^5^	584	178	0.56	7.83
**5a**	540	4.0 × 10^4^	569	944	0.25	5.70
**6a**	578	6.3 × 10^4^	597	551	0.16	5.52
**1b**	502	8.8 × 10^4^	510	312	0.96	5.35
**5b**	529	7.8 × 10^4^	547	622	0.86	5.59
**6b**	558	9.7 × 10^4^	574	500	0.77	4.97

^a^Absorption coefficients of the S_0_–S_1_ bands in CH_2_Cl_2_. The reference values are taken from the literature [32,43]. ^b^Emission maxima at wavelength. ^c^Emission quantum yield determined by integrating sphere. ^d^Emission lifetime probed at the maxima of the bands.

In the case of β-substituted BODIPYs, the S_0_–S_1_ absorption bands of **5a** and **6a** are significantly broadened with the full width at half maximum (fwhm) of 1827 and 1438 cm^−1^, respectively, compared to those of the α-substituted compounds **3a** and **4a** (625 and 508 cm^−1^, respectively, Figure 4a). Accordingly, the relatively broad emission bands were observed with lowered quantum yields, Φ_f_ of 0.25 and 0.16, respectively (Figure 5b and Table 1). The larger Stokes shift and shorter emission lifetime suggest a rapid decay of the excited species after structural relaxation (vide infra, Table 1). Also, intersystem crossing to the triplet state can be another pathway to understand the lower emission quantum yield of the BODIPYs. However, reactive singlet oxygen species (^1^O_2_) was not observed under the aerobic conditions probed by the near-infrared photoluminescence at 1270 nm. The above spectral features were likewise shown with the β-ethylene-substituted BODIPYs [32].

Similarly, the bathochromic shifts of the absorption and fluorescence bands for the tetramethyl-substituted derivatives **5b** and **6b** were observed in CH_2_Cl_2_ solution (Figure S21, Supporting Information File 1). However, due to the rigid structure of the highly substituted BODIPY cores, higher emission quantum yields of 0.86 and 0.77 were estimated, respectively (Table 1).

### Electrochemical properties

Cyclic voltammetry and differential pulse voltammetry of the BODIPY derivatives **3a**–**6a** were performed in dichloromethane solutions containing 0.1 M tetra-*n*-butylammonium hexafluorophosphate (TBAPF_6_) as a supporting electrolyte (Figure S23, Supporting Information File 1). All the derivatives exhibited reversible one-electron reduction waves and the irreversible oxidation ones, except for **4a**. Upon installing the TIPS-ethynyl substituents into the BODIPY core, the reduction potentials are significantly shifted in the anodic direction rather than the extent of the shifts in the oxidation potentials [30]. The electron-deficient effect of the substituents is pronounced for the doubly substituted derivatives **4a** and **6a**. Consequently, the electrochemical energy gaps of **3a**–**6a** are estimated to be 2.30, 2,14, 2.35, and 2.21 V, respectively, which is consistent with the calculated HOMO–LUMO energy gaps (vide infra, Figure S24, Supporting Information File 1).

### Theoretical calculations

To gain insight into the substitution effect on the electronic properties of the BODIPY derivatives, density functional theory (DFT) calculations were performed at the B3LYP/6-31G(d) level of theory. The *a*_2_-symmetry of the HOMOs and *b*_2_-symmetry of the LUMOs of each, the α- and β-ethynyl-substituted BODIPYs are almost identical to those of the unsubstituted compound **1a** (Figure S24, Supporting Information File 1). The HOMO energies of the α-ethynyl-substituted derivatives **3a** and **4a**, are destabilized with broken degeneracy upon increasing the number of ethynyl substituents. On the other hand, the corresponding energies of the β-substituted derivatives **5a** and **6a** are not drastically altered because of a nodal plane near the 1,7-positions in the HOMO and smaller MO coefficients at the 2,6-positions than those at the 3,5-positions. Rather, a stabilization of the LUMO energies is found in **5a** and **6a** even though the MO hybridization of the BODIPY core with π-orbitals of ethynyl moieties is less affected. The mesomeric effect on the ethynyl substituents at the β-position of the BODIPY core is thus different from the α-substituted ones.

Furthermore, to understand the excited-state properties (e.g., Stokes shifts) of the substituted BODIPYs, the S_1_-state geometries for **4a** and **6a** were obtained by the time-dependent (TD) DFT methods. As a result, the excited-state structure of **6a** exhibits a remarkably bent distortion of the core with a plane angle of 16.1°, whereas the structure of **4a** remains coplanar (Figure S25, Supporting Information File 1). The corresponding energy gap between the HOMO and LUMO of **6a** is significantly decreased with the geometry relaxation at the S_1_ state compared to that of the S_0_-state structure, which could be the origin of the large Stokes shift for **6a**. Therefore, in comparison with the α-substituted compounds **3a** and **4a**, the less emissive character of the β-ethynyl-substituted derivatives **5a** and **6a** agreed with the fact that the photoexcited dynamics of β-substituted BODIPYs intend to the rapid decay with structural relaxation.

## Conclusion

In summary, we have synthesized novel regioselectively alkynylated BODIPY derivatives via a gold(I)-catalyzed direct C–H functionalization with TIPS-EBX reagents. This atom-economical method for the late-stage alkynylation of BODIPY dyes could be an alternate approach for functionalized fluorescent dyes without the need of preparing unstable halogenated pyrrole precursors. The resulting α- and β-ethynyl-substituted BODIPYs displayed distinct substitution-site-dependent spectral features, for instance, the extent of the bathochromic shifts of the absorption and fluorescence, variable Stokes shift and the emission quantum yields. The TIPS-protected ethynyl groups of these BODIPY dyes can be applied as substrates for the “click” CuAAC reaction toward novel functional fluorescent materials.

## Supporting Information

File 1 Experimental methods including detailed synthetic procedures, compound characterization data, and DFT calculations.

File 2 Crystallographic information file of compound **3a**.

File 3 Crystallographic information file of compound **6b**.
