# Supplementary material for: Regioselectively α- and β-alkynylated BODIPY dyes via gold(I)-catalyzed direct C–H functionalization and their photophysical properties
Source: Beilstein J Org Chem. 2020 Apr 1;16:587–95. doi: 10.3762/bjoc.16.53 (PMC7136566; doi:10.3762/bjoc.16.53)
Supplement: File 1 — Experimental methods including detailed synthetic procedures, compound characterization data, and DFT calculations. [file Beilstein_J_Org_Chem-16-587-s001.pdf]

BEILSTEIN JOURNAL OF ORGANIC CHEMISTRY

Supporting Information

for

Regioselectively α- and β-alkynylated BODIPY dyes via gold(I)-catalyzed direct C–H functionalization and their photophysical properties

Takahide Shimada, Shigeki Mori, Masatoshi Ishida and Hiroyuki Furuta

Beilstein J. Org. Chem. **2020,** *16,* 587–595. doi:10.3762/bjoc.16.53

Experimental methods including detailed synthetic procedures, compound characterization data, and DFT calculations

License and Terms: This is a supporting information file under the terms of the Creative Commons Attribution License (http://creativecommons.org/licenses/by/4.0). Please note that the reuse, redistribution and reproduction in particular requires that the authors and source are credited.
The license is subject to the *Beilstein Journal of Organic Chemistry* terms and conditions: (https://www.beilstein-journals.org/bjoc)

Table of contents

1. General information S2
2. Synthesis and compound data S3
3. ^{1}H, ^{11}B, ^{19}F NMR spectra S8
4. X-ray crystallography S18
5. Photophysical properties S20
6. Electrochemical properties S22
7. DFT calculation S23
8. References S25
9. Cartesian coordinates S26

1 General information

Materials:

Commercially available solvents and reagents were used without further purifications unless otherwise mentioned. Preparative separation was performed by silica gel column chromatography on KANTO silica gel 60 N, spherical, neutral, 40–50 μm. Thin-layer chromatography (TLC) was carried out on aluminum sheets coated with silica gel 60 F_{254} (MERCK). 5-Mesityl BODIPY (**1a**), 8-mesityl-1,3,5,7-tetramethyl BODIPY (**1b**) and TIPS-EBX can be synthesized using reported procedures [S1–S3].

Instrumentals:

^{1}H, ^{11}B, and ^{19}F NMR were recorded on a JEOL ECZ-500R spectrometer. Chemical shifts (δ) are reported in ppm relative to residual solvent ($CDCl_3$: 7.26 ppm for ^{1}H). Boron trifluoride ethyl ether complex was used as external reference for ^{11}B (δ = 0.00 ppm). Trifluoroacetic acid was used as external reference for ^{19}F (δ = −76.5 ppm). UV–vis–NIR spectra were measured on a Shimadzu UV-3150PC spectrometer. Fluorescence spectra were recorded on an SPEX Fluorolog-3-NIR spectrometer (HORIBA) with NIR-PMT R5509 photomultiplier tube (Hamamatsu). High resolution mass (HRMS) spectra were obtained in fast atom bombardment (FAB mode) with 3-nitrobenzyl alcohol (NBA) as a matrix on a JEOL LMS-HX-110 spectrometer. The absolute photoluminescence quantum yields (Φ_f) were determined using absolute PL quantum yields measurement system C9920-02 (Hamamatsu photonics). Time-resolved photoluminescence lifetimes were carried out by using time-correlated single-photon counting lifetime spectroscopy system, Quantaurus-Tau C11367-02 (Hamamatsu photonics). The decay constants and fitting parameters for transient decays were determined using the embedded software of Quantaurus-Tau. Cyclic voltammograms and differential pulse voltammograms were recorded on a CH Instrument Model 620B (ALS) under argon atmosphere in a dichloromethane solution with 0.1 M tetra-*n*-butylammonium hexafluorophosphate as supporting electrolyte. Measurements were performed with a glassy carbon working electrode, an Ag/AgCl reference electrode, and a Pt wire counter electrode. The concentration of the solution was fixed at 1 mM, and the sweep rates were set to 100 mV s^{-1}. The ferrocenium/ferrocene (Fc^{+}/Fc) couple was used as an internal standard.

X-ray crystallographic analysis:

X-ray analysis was performed on a SMART APEX equipped with a CCD detector (Bruker) using Mo K_α (graphite, monochromated, λ = 0.71069 Å) radiation. The structures were solved by the direct method of SHELXT 2014/5 and refined using the SHELXL-2016/6 program [S4]. The positional parameters and thermal parameters of non-hydrogen atoms were refined anisotropically on F^2 by the full-matrix least-squares method. Hydrogen atoms were placed at calculated positions

and refined riding on their corresponding carbon atoms. Details of the structures and their refinement may be obtained from the Cambridge Crystallographic Data Centre.

Calculation details:

Theoretical calculations were performed with the Gaussian16 program package [S5]. All calculations were carried out by the density functional theory (DFT) method with the Becke's three-parameter hybrid exchange functional and the Lee-Yang-Parr correlation functional (B3LYP), employing a basis set containing 6-31G(d) for all atoms [S6]. The X-ray crystallographic structures were used as initial geometries for geometry optimization without symmetry restrictions. Geometry optimization in the S_1 states was performed by time-dependent (TD)-DFT method. The geometries were fully optimized and verified by the frequency calculations, where no imaginary frequency was found.

2 Synthesis and compounds data

Synthesis of 8-mesityl-3-(triisopropylsilyl)ethynyl BODIPY (3a)

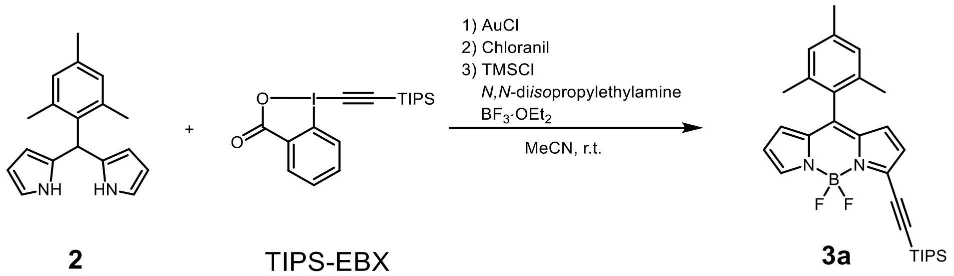

5-Mesityl-substituted dipyrromethane (**2**, 100 mg, 0.38 mmol) was added to a mixture solution of AuCl (4.8 mg, 5 mol %) and TIPS-EBX (161 mg, 0.38 mmol) in MeCN (5.0 mL) under air. The reaction vessel was sealed and the solution stirred at room temperature for 16 h. Then, *p*-chloranil (93.6 mg, 0.38 mmol, 1.0 equiv) was added and the resulting solution was stirred for 4 h. Trimethylsilyl chloride (TMSCl, 1.0 mL, 7.6 mmol), *N,N*-diisopropylethylamine (1.0 mL, 5.7 mmol) and $BF_3 \cdot OEt_2$ (0.90 mL, 6.9 mmol) were added and the reaction mixture was stirred for 2 h. Finally, the reaction was quenched by the addition of aq. $NaHCO_3$. The product was extracted with CH_2Cl_2. The organic layer was dried over Na_2SO_4 and concentrated in vacuo. The residue was purified by silica gel column chromatography (AcOEt/hexane) to give a reddish solid of **3a**.

Yield 32 mg (18%). ^{1}H NMR (500 MHz, $CDCl_3$): δ = 7.95 (s, 1H), 6.94 (s, 2H.), 6.65 (d, 1H, J = 4.5 Hz), 6.57 (t, 2H, J = 4.5 Hz), 6.47 (d, 1H, J = 4.0 Hz), 2.35 (s, 3H), 2.07 (s, 6H), 1.18 (s, 21H); ^{19}F NMR (470 MHz, $CDCl_3$): δ = –147.26 (m); ^{11}B NMR (160 MHz, $CDCl_3$): δ = 0.34 (t, J = 2.9 Hz); HR-FAB-MS: calcd: 490.2793 for $C_{29}H_{37}BF_2N_2Si$, found: 490.2800 (Err +2.6 ppm)

Synthesis of 8-mesityl-3,5-bis((triisopropylsilyl)ethynyl) BODIPY (4a)

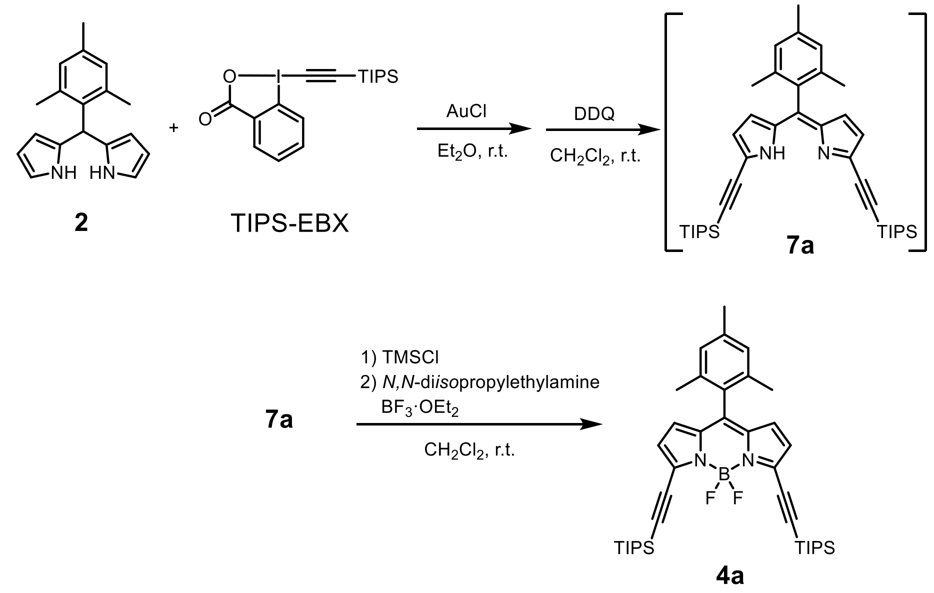

5-Mesityl-substituted dipyrromethane (**2**, 110 mg, 0.40 mmol) was added to a mixture solution of AuCl (4.7 mg, 2 mol %) and TIPS-EBX (380 mg, 0.88 mmol) in Et_2O (8.0 mL) under air. The reaction vessel was sealed and the solution was stirred at room temperature for 16 h. Then, Et_2O (8.0 mL) and 0.1 M aq. NaOH were added to the mixture. The organic layer was washed with aqueous $NaHCO_3$, dried over Na_2SO_4 and concentrated in vacuo. DDQ (88 mg, 0.40 equiv) in CH_2Cl_2 (4 mL) was added to the mixture and stirred for 25 min. After removal of the solvents in vacuo, the residue was purified by Al_2O_3 column chromatography (CH_2Cl_2/hexane) to give dipyrrin **7a** (roughly 95 mg). The dipyrrin is not stable under ambient conditions. Subsequently, the ethynyl-substituted dipyrrin (**7a**, 12 mg, 0.018 mmol) was dissolved in CH_2Cl_2 (4.0 mL) and complexed with $BF_3{\cdot}OEt_2$ (0.037 mL, 0.29 mmol) in the presence of TMSCl (0.041 mL, 0.32 mmol) and *N,N*-diisopropylethylamine (0.042 mL, 0.24 mmol) with stirring at room temperature for 1 h. The resulting mixture was extracted with aq. $NaHCO_3$ and CH_2Cl_2, and dried over Na_2SO_4. After removal of the solvent under reduced pressure, the residue was purified by silica gel column chromatography (CH_2Cl_2/hexane) and recrystallization from CH_2Cl_2/methanol.

Yield: 16% (in three steps). ^{1}H NMR (500 MHz, $CDCl_3$): δ = 6.92 (s, 2H), 6.54 (d, 2H, J = 5.0 Hz), 6.53 (d, 2H, J = 4.5 Hz), 2.33 (s, 3H), 2.05 (s, 6H), 1.16 (s, 42H); ^{19}F NMR (470 MHz, $CDCl_3$): δ = –147.77 (m); ^{11}B NMR (160 MHz, $CDCl_3$): δ = 0.54 (t, J = 2.7 Hz); HR-FAB-MS: calcd: 670.41219 for $C_{40}H_{57}BF_2N_2Si_2$, found: 670.4116 (Err –0.8 ppm).

^{1}H NMR data for **7a:** ^{1}H NMR (500 MHz, $CDCl_3$): δ = 6.90 (s, 2H), 6.42 (d, 2H, J = 4.5 Hz), 6.29 (d, 2H, J = 4.0 Hz), 2.33 (s, 3H), 1.99 (s, 6H), 1.11 (br s, 42H; TIPS).

Synthesis of 8-mesityl-2-(triisopropylsilyl)ethynyl BODIPY (5a) and 8-mesityl-2,6-bis((triisopropylsilyl)ethynyl) BODIPY (6a)

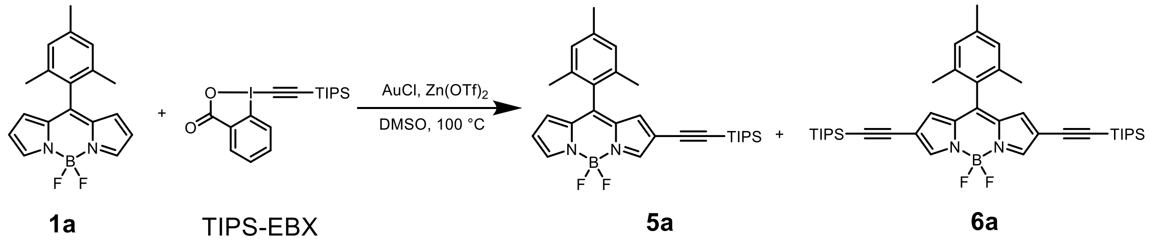

8-Mesityl BODIPY (**1a**, 31 mg, 0.10 mmol) was added to a mixture solution of AuCl (2.2 mg, 10 mol %), TIPS-EBX (93.3 mg, 0.22 mol), and $Zn(OTf)_2$ (81.4 mg, 0.22 mol) in DMSO (3.0 mL) under air. The reaction vessel was sealed and the solution was stirred at 100 °C for 1.5 days. The resulting product was washed with aq. $NaHCO_3$ and extracted with CH_2Cl_2. The organic layer was dried over Na_2SO_4 and concentrated in vacuo. The residue was purified by silica gel column chromatography (CH_2Cl_2/hexane) to give two corresponding fractions containing monosubstituted **5a** and disubstituted **6a**, respectively.

Yield for **5a**, 18 mg (38%). ^{1}H NMR (500 MHz, $CDCl_3$): δ = 7.97 (s, 1H), 7.96 (s, 1H), 6.95 (s, 2H), 6.72 (d, 2H, J = 4.5 Hz), 6.67 (s, 1H), 6.50 (d, 1H, J = 4.0 Hz), 2.36 (s, 3H), 2.10 (s, 6H), 1.07 (s, 21H); ^{19}F NMR (470 MHz, $CDCl_3$): δ = –146.54 (m); ^{11}B NMR (160 MHz, $CDCl_3$): δ = 0 (t, J = 2.9 Hz); HR-FAB-MS: calcd: 490.2793 for $C_{29}H_{37}BF_2N_2Si$, found: 490.2784 (Err –0.6 ppm).

Spectral data of **5a** are identical to the literature data [S7].

Yield for **6a**, 1.4 mg (2%). ^{1}H NMR (500 MHz, $CDCl_3$): δ = 7.99 (s, 2H), 6.95 (s, 2H), 6.69 (s, 2H), 2.35 (s, 3H), 2.10 (s, 6H), 1.15 (m, 42H); ^{19}F NMR (470 MHz, $CDCl_3$): δ = –146.47 (m); ^{11}B NMR (160 MHz, $CDCl_3$): δ = 0 (t, J = 2.9 Hz); HR-FAB-MS: calcd: 670.4129 for $C_{40}H_{57}BF_2N_2Si_2$, found: 670.4118 (Err –0.5 ppm).

We have tried to optimize the reaction conditions for the ethynyl-substituted BODIPYs **5a** and **6a** as shown in the Table S1. The detailed procedure is given above.

Table S1: Optimization of the alkynylation of BODIPY (**1a**).

Entry	Solvent	Additive	Temperature (°C)	Yield (%) **5a**	Yield (%) **6a**
1	CH_2Cl_2	none	r.t	none	None
2	THF	none	70	trace	None
3	MeCN	TFA	60	9.5 (18)[a]	1.0 (1.8)[a]
4	MeCN	TFA	75	12 (28)[a]	Trace
5	MeCN	$Zn(OTf)_2$	60	21 (48)[a]	0.5 (1)[a]
6	DMSO	$Zn(OTf)_2$	100	38 (66)[a]	2.1 (4)[a]
7	DMSO	$Zn(OTf)_2$	120	12 (14)[a]	3.0 (4)[a]

[a]Yields given in parentheses are estimated based on the converted BODIPY **1a**.

Synthesis of 8-mesityl-1,3,5,7-tetramethyl-2-(triisopropylsilyl)ethynyl BODIPY (5b) and 8-mesityl-1,3,5,7-tetramethyl-2,6-bis((triisopropylsilyl)ethynyl) BODIPY (6b)

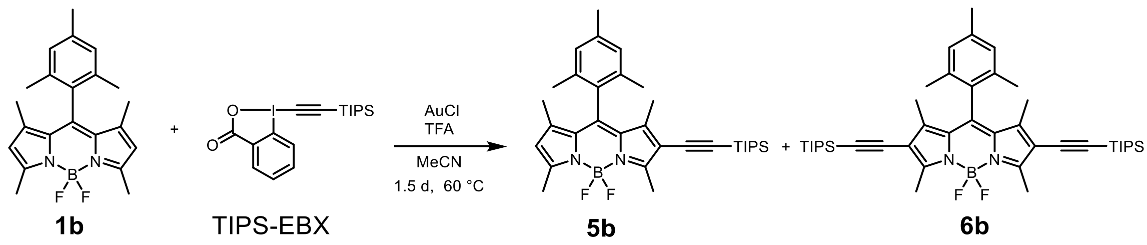

8-Mesityl-1,3,5,7-tetramethyl BODIPY (**1b**, 20 mg, 0.055 mmol) was added to a mixture solution of AuCl (1.3 mg, 10 mol %) and TIPS-EBX (51.8 mg, 0.12 mol) in MeCN (3.0 mL) under air. After the addition of TFA (4.5 µL, 0.055 mmol), the reaction vessel was sealed and the solution was stirred at

60 °C for 1.5 days. The reaction mixture was washed with aq. $NaHCO_3$, and extracted with CH_2Cl_2. The organic layer was dried over Na_2SO_4 and concentrated in vacuo. The residue was purified by silica gel column chromatography (CH_2Cl_2/hexane) to give the corresponding products, **5b** and **6b**, respectively.

Yield for **5b**, 7.7 mg (26%). ^{1}H NMR (500 MHz, $CDCl_3$): δ = 6.95 (s, 2H), 6.01 (s, 1H), 2.65 (s, 3H.), 2.57 (s, 3H), 2.33 (s, 3H), 2.08 (s, 6H), 1.49 (s, 3H), 1.40 (s, 3H), 1.09 (s, 21H); ^{19}F NMR (470 MHz, $CDCl_3$): δ = −147.47 (q); ^{11}B NMR (160 MHz, $CDCl_3$): δ = 0 (t, J = 3.4 Hz); HR-FAB-MS: calcd: 546.3413 for $C_{33}H_{45}BF_2N_2Si_1$, found: 546.3419 (Err −0.6 ppm).

Yield for **6b**, 7.4 mg (19%). ^{1}H NMR (500 MHz, $CDCl_3$): δ = 6.95 (s, 2H), 2.65 (s, 6H.), 2.33 (s, 3H), 2.07 (s, 6H), 1.55 (s, 6H), 1.50 (s, 6H), 1.09 (s, 42H). ^{19}F NMR (470 MHz, $CDCl_3$): δ = −147.52 (q); ^{11}B NMR (160 MHz, $CDCl_3$): δ = 0.46 (t, J = 3.2 Hz); HR-FAB-MS: calcd: 726.4747 for $C_{44}H_{65}BF_2N_2Si_2$, found: 726.4756 (Err +0.4 ppm).

3 ^{1}H, ^{11}B, ^{19}F NMR Spectra

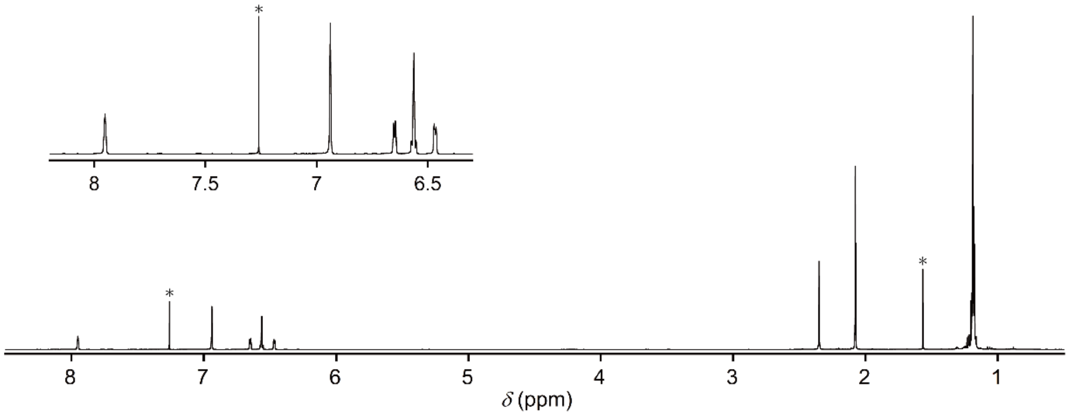

Figure S1: ^{1}H NMR spectrum of **3a** in $CDCl_3$ at 298 K. Asterisk indicates the residual solvent peaks.

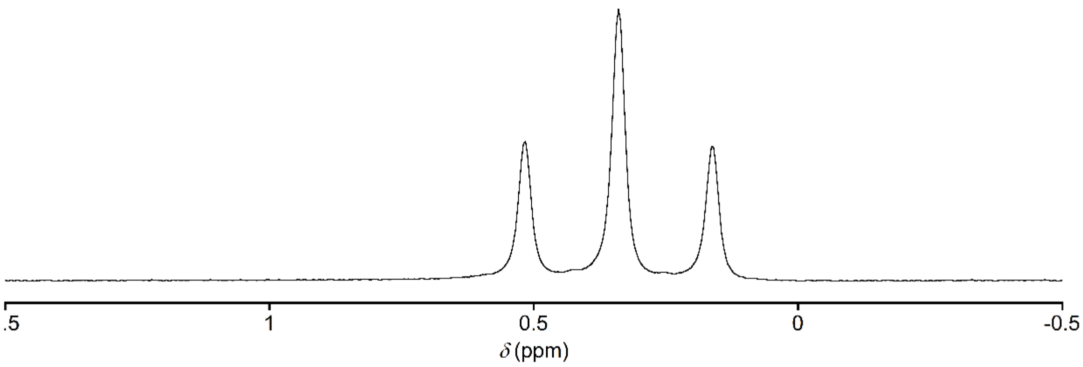

Figure S2: ^{11}B NMR spectrum of **3a** in $CDCl_3$ at 298 K.

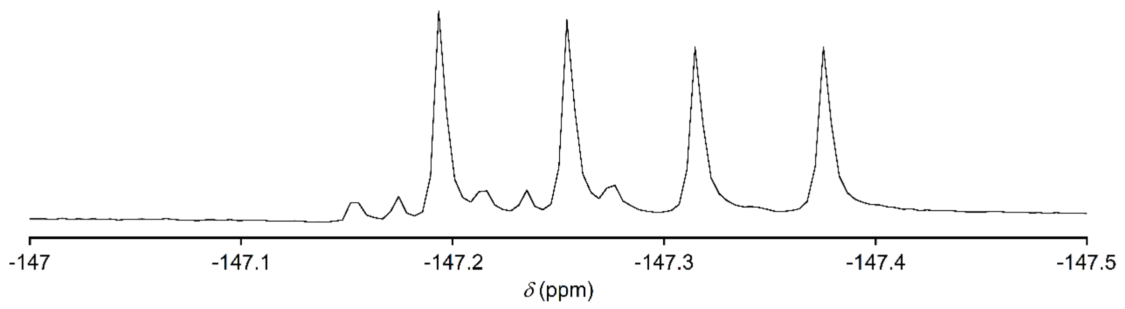

Figure S3: ^{19}F NMR spectrum of **3a** in $CDCl_3$ at 298 K.

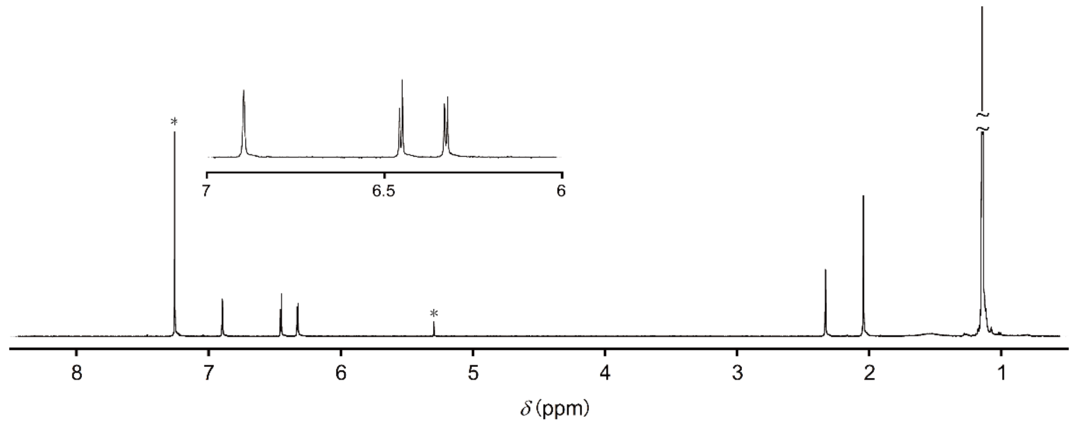

Figure S4: ^{1}H NMR spectrum of **7a** in $CDCl_3$ at 298 K. Asterisk indicates the residual solvent peaks.

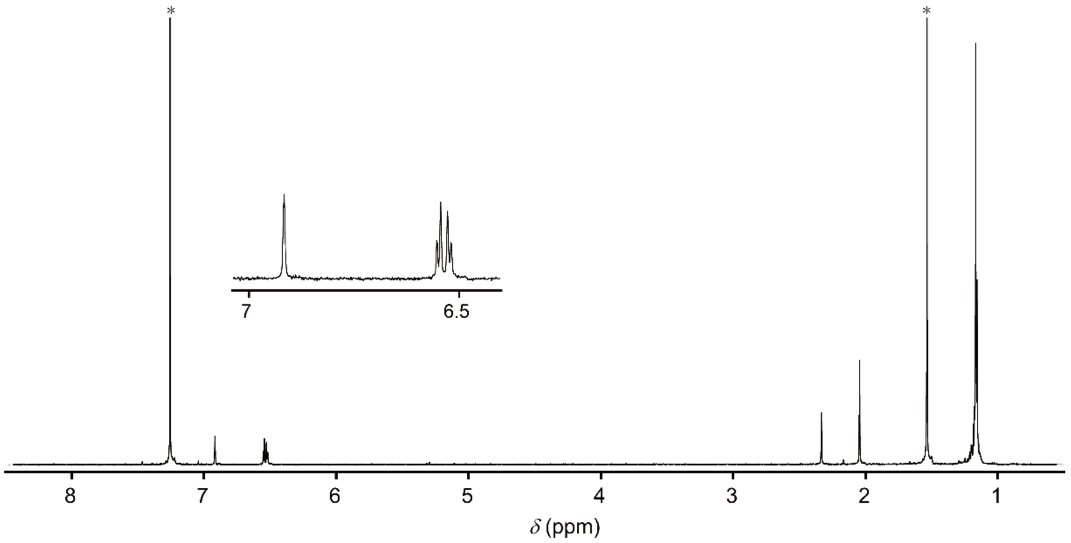

Figure S5: ^{1}H NMR spectrum of **4a** in $CDCl_3$ at 298 K. Asterisk indicates the residual solvent peaks.

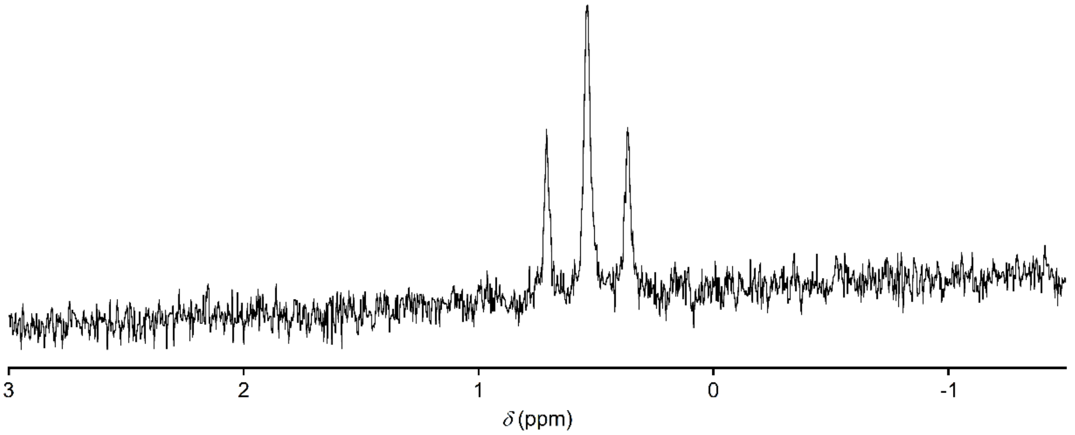

Figure S6: ^{11}B NMR spectrum of **4a** in $CDCl_3$ at 298 K.

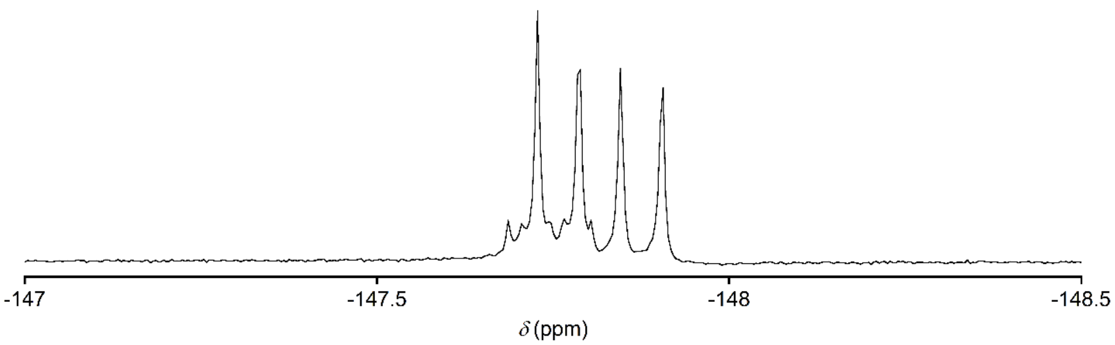

Figure S7: ^{19}F NMR spectrum of **4a** in $CDCl_3$ at 298 K.

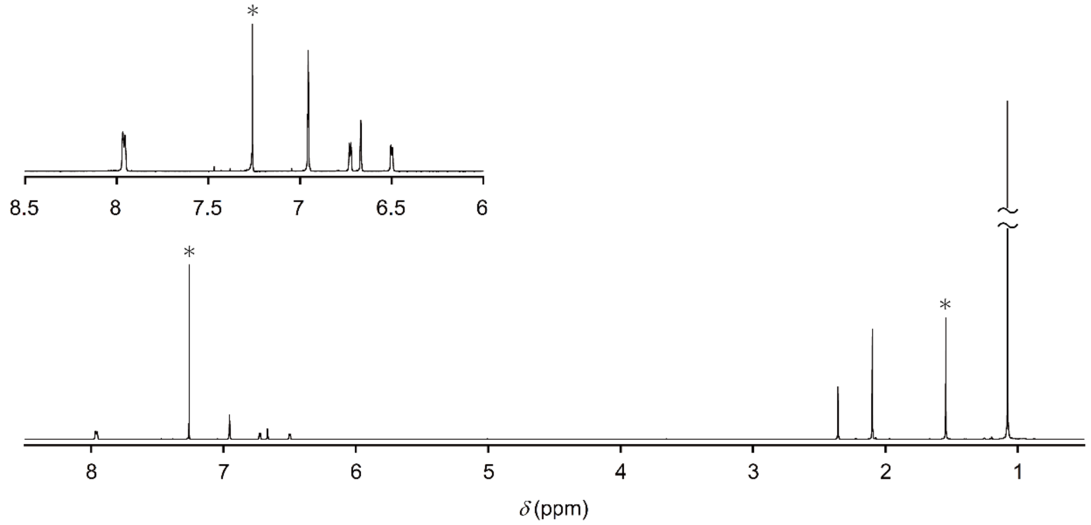

Figure S8: ^{1}H NMR spectrum of **5a** in $CDCl_3$ at 298 K. Asterisk indicates the residual solvent peaks.

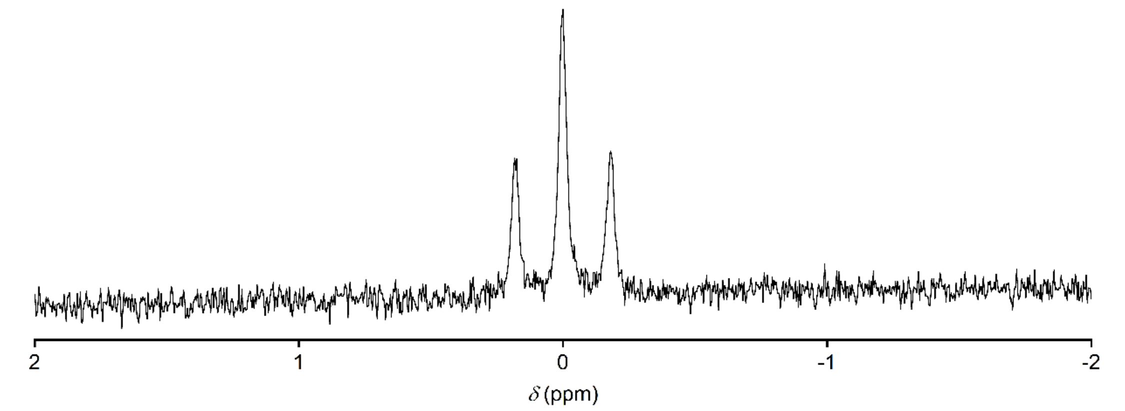

Figure S9: ^{11}B NMR spectrum of **5a** in $CDCl_3$ at 298 K.

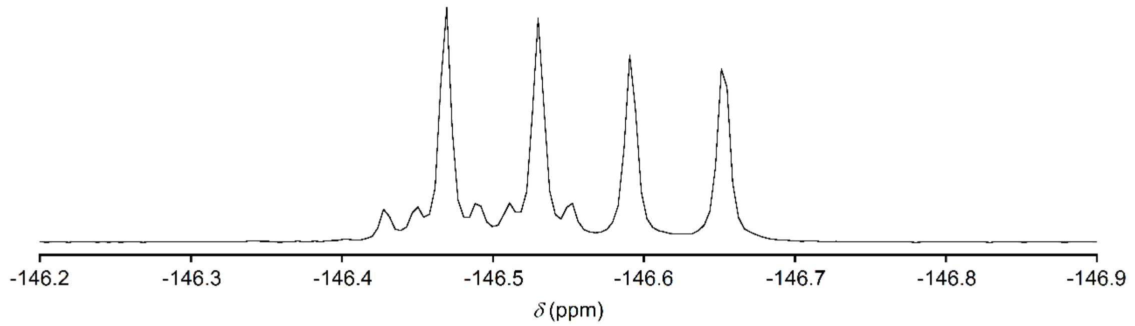

Figure S10: ^{19}F NMR spectrum of **5a** in $CDCl_3$ at 298 K.

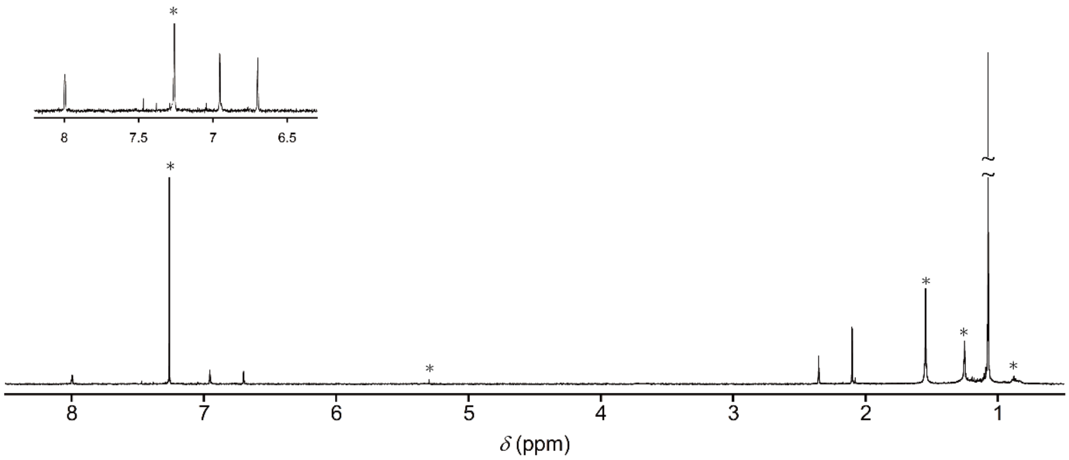

Figure S11: ^{1}H NMR spectrum of **6a** in $CDCl_3$ at 298 K. Asterisk indicates the residual solvent peaks.

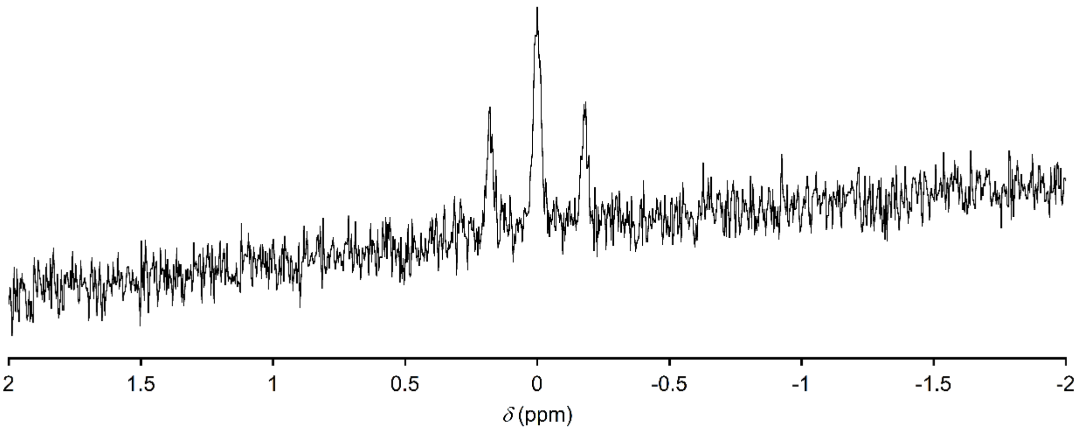

Figure S12: ^{11}B NMR spectrum of **6a** in $CDCl_3$ at 298 K.

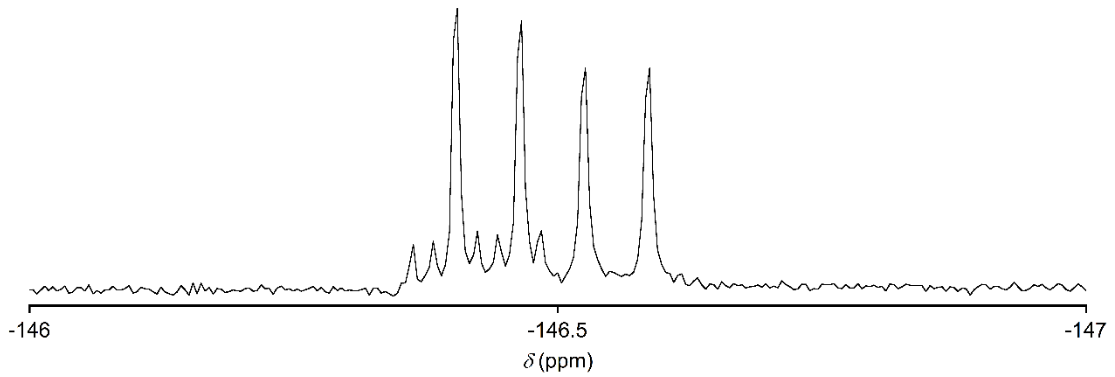

Figure S13: ^{19}F NMR spectrum of **6a** in $CDCl_3$ at 298 K.

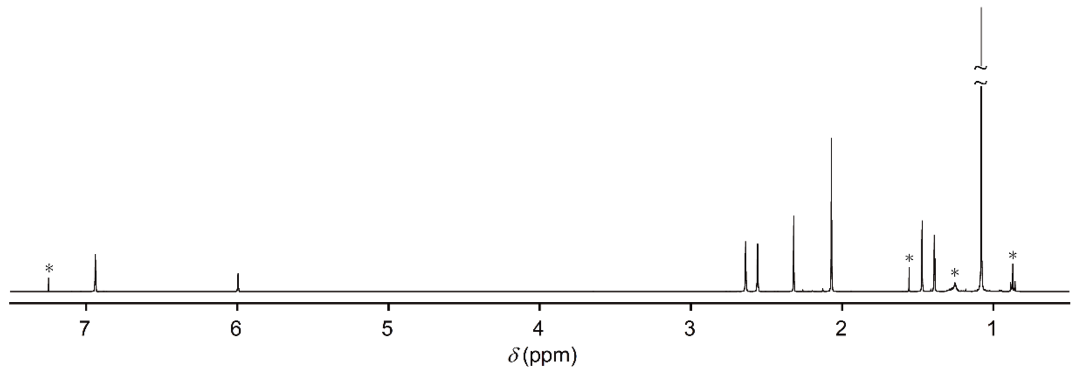

Figure S14: ^{1}H NMR spectrum of **5b** in $CDCl_3$ at 298 K. Asterisk indicates the residual solvent peaks.

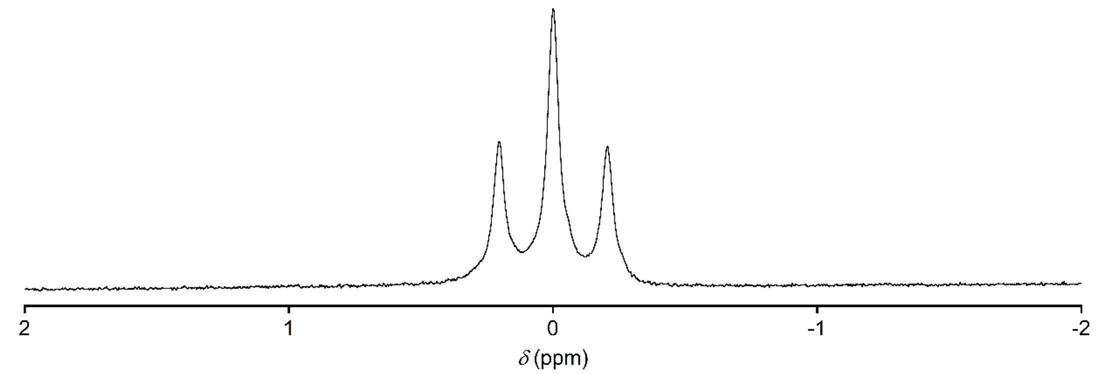

Figure S15: ^{11}B NMR spectrum of **5b** in $CDCl_3$ at 298 K.

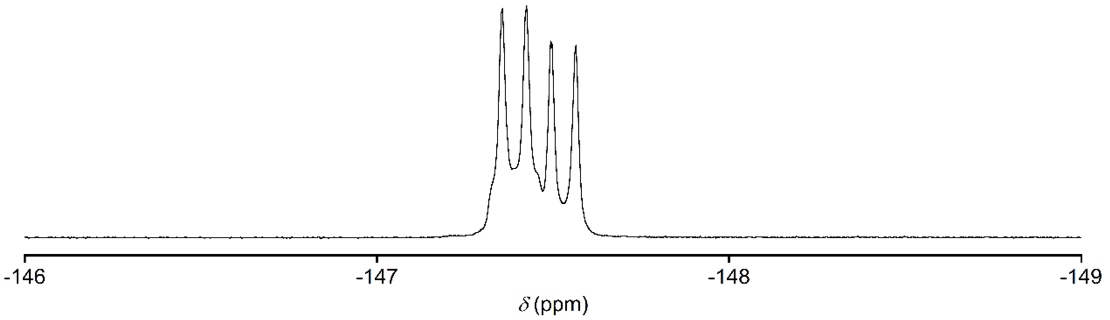

Figure S16: ^{19}F NMR spectrum of **5b** in $CDCl_3$ at 298 K.

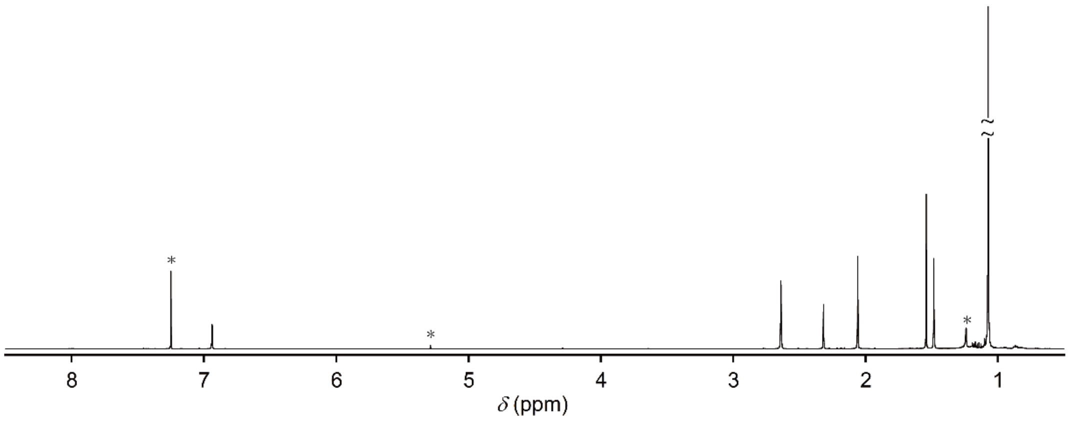

Figure S17: ^{1}H NMR spectrum of **6b** in $CDCl_3$ at 298 K. Asterisk indicates the residual solvent peaks.

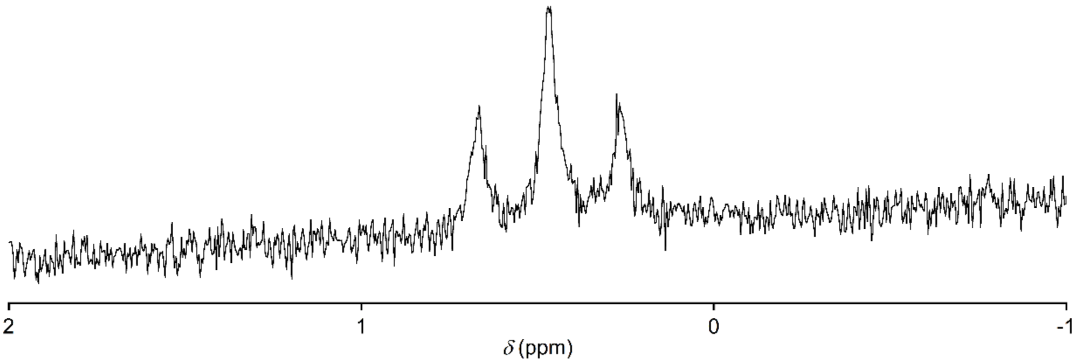

Figure S18: ^{11}B NMR spectrum of **6b** in $CDCl_3$ at 298 K.

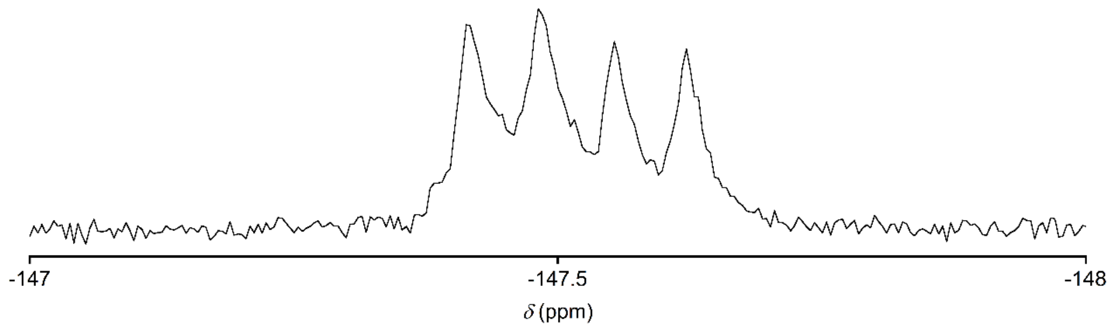

Figure S19: ^{19}F NMR spectrum of **6b** in $CDCl_3$ at 298 K.

4 X-ray crystallography

Table S2: Crystallographic data of **4a** and **6b**.

	**4a**	**6b**
Empirical Formula	$C_{40}H_{57}BF_2N_2Si_2$	$C_{44}H_{65}BF_2N_2Si_2$
Formula Weight	670.88	726.99
Temperature	–173.0 °C	–173.0 °C
Crystal Color, Habit	green, block	red, plate
Crystal Dimensions	0.250 x 0.200 x 0.180 mm	0.170 x 0.150 x 0.010 mm
Crystal System	monoclinic	monoclinic
Lattice Parameters	a = 19.9534(4) Å	a = 33.9498(8) Å
	b = 8.04654(17) Å	b = 21.4322(5) Å
	c = 25.0121(5) Å	c = 12.1761(3) Å
	β = 101.750(2)°	β = 100.114(2)°
	V = 3931.69(14) $Å^3$	V = 8721.9(4) $Å^3$
Space Group	$P2/n$ (#13)	$C2$ (#5)
Z value	4	8
D_{calc}	1.133 g/cm^3	1.107 g/cm^3
F_{000}	1448	3152
μ(MoKα)	1.285 cm^{-1}	1.205 cm^{-1}
R_1	0.0462	0.0640
wR_2	0.1174	0.1603
$2\theta_{max}$	56.0°	56.0°
CCDC	1978927	1978925

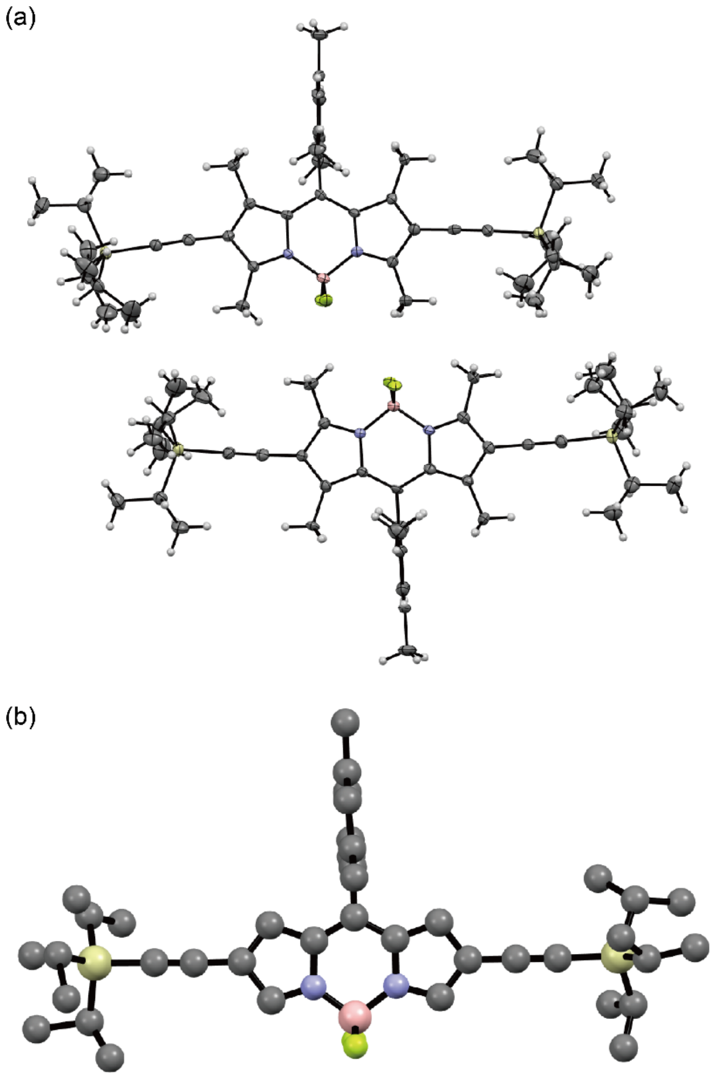

Figure S20: (a) Two independent X-ray crystal structures of **6b** found in the lattice and (b) the preliminary crystal structure of **6a**.

5 Photophysical properties

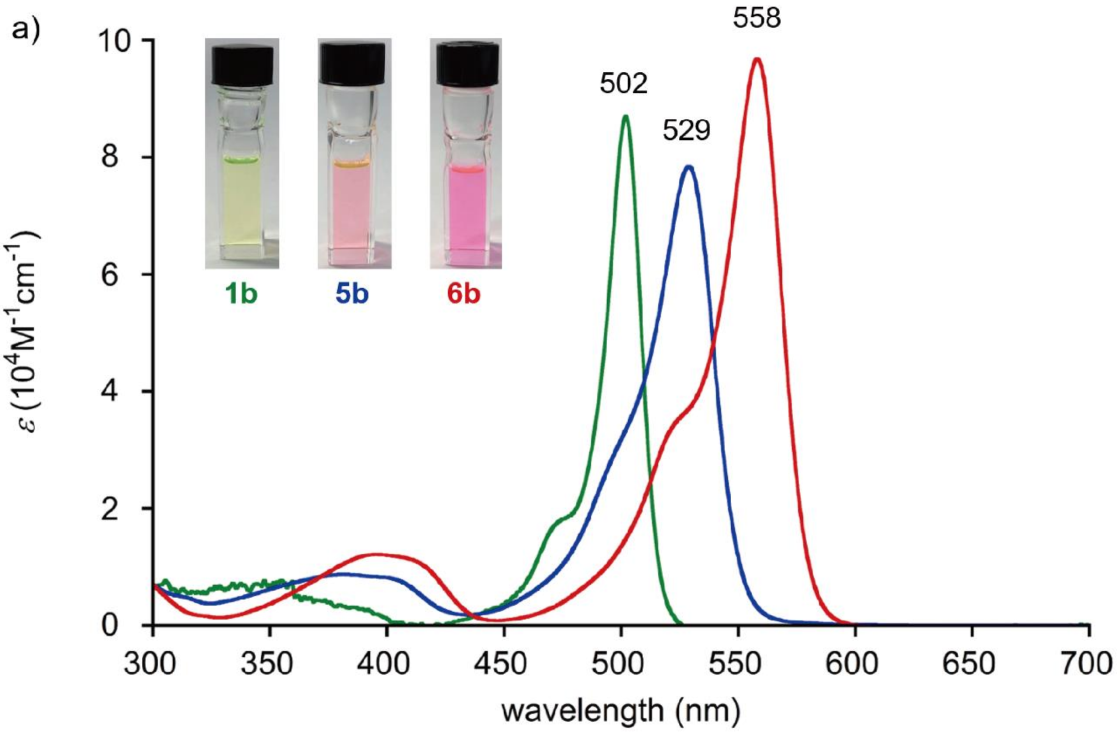

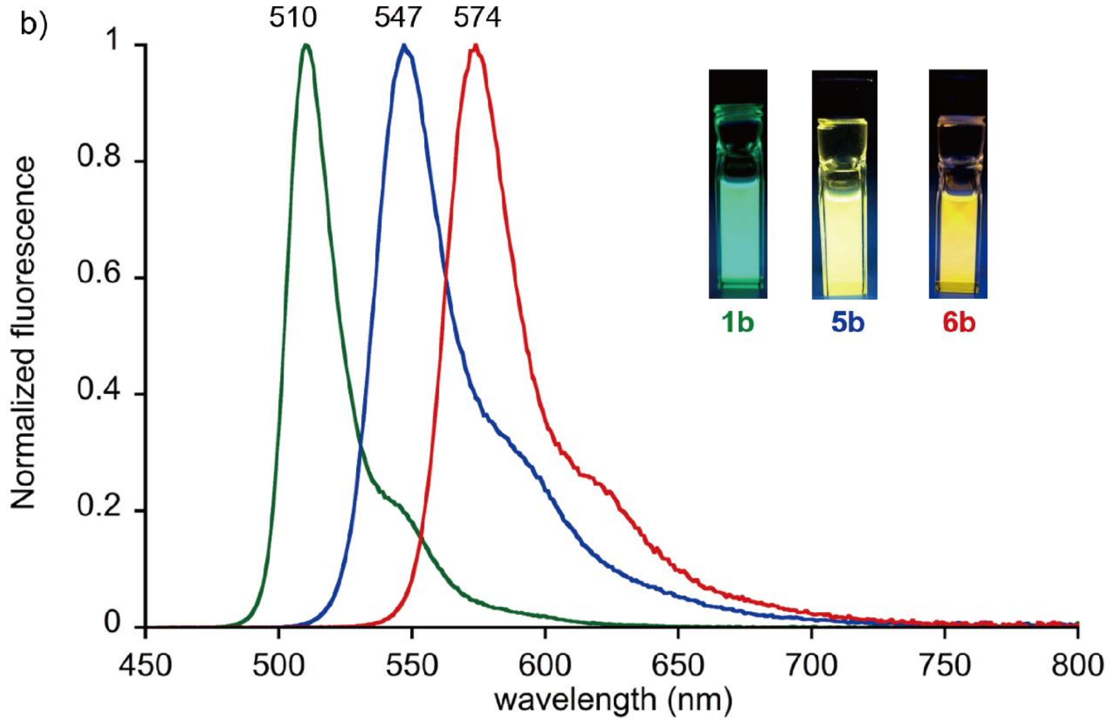

Figure S21: (a) UV–vis absorption and (b) fluorescence spectra of BODIPY derivatives, **1b** (green), **5b** (blue), and **6b** (red) in CH_2Cl_2. Insets show the photo images of the solution taken under (a) ambient light and (b) UV lamp, respectively.

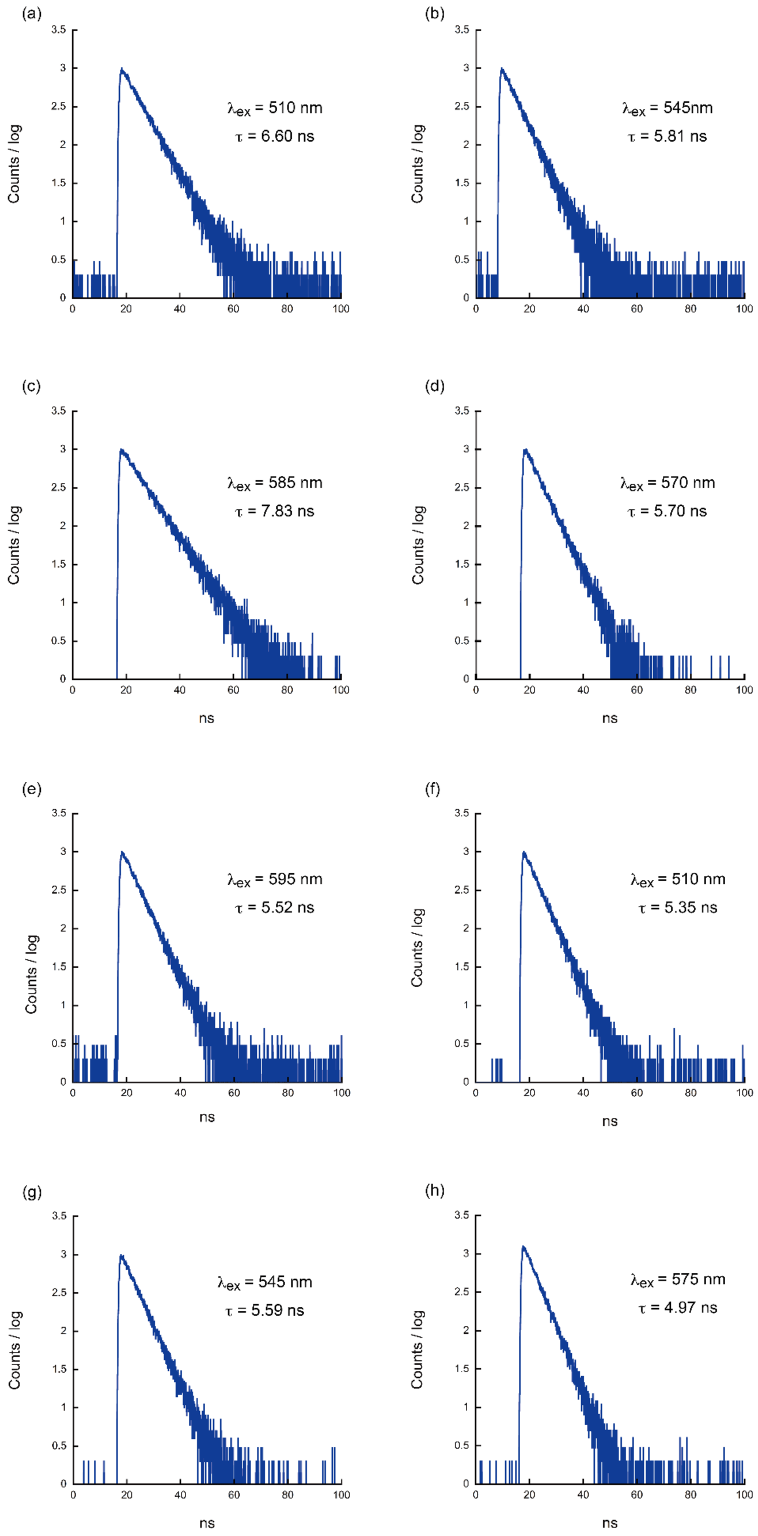

Figure S22: Fluorescence lifetime of BODIPY derivatives, (a) **1a**, (b) **3a**, (c) **4a**, (d) **5a**, (e) **6a**, (f) **1b**, (g) **5b**, and (h) **6b** in CH_2Cl_2. Excitation was performed at 365 nm.

6 Electrochemical properties

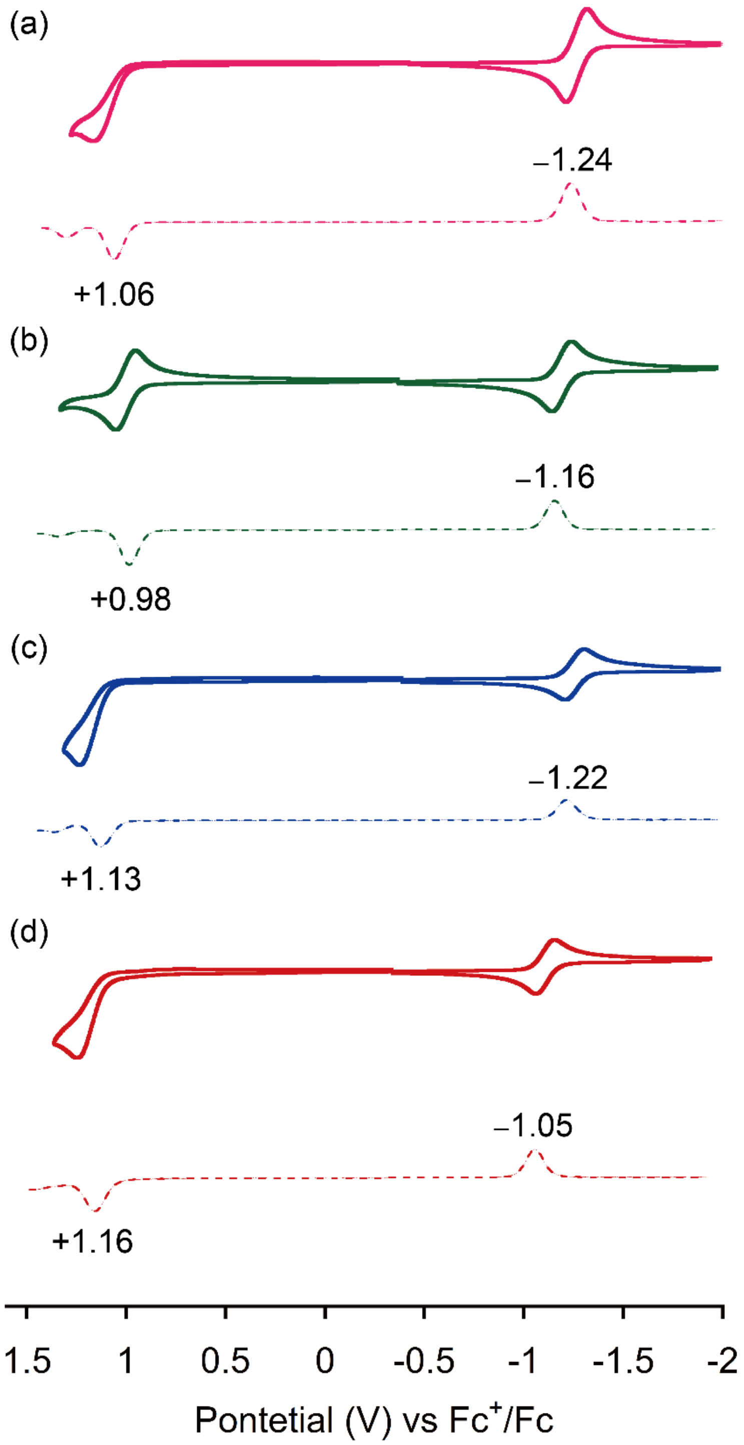

Figure S23: Cyclic voltammograms (solid line) and differential pulse voltammograms (dashed line) of (a) **3a**, (b) **4a**, (c) **5a,** and (d) **6a**, measured in dichloromethane solution containing 0.1 M $TBAPF_6$ as the supporting electrolyte. Scan rate is 0.1 V/s.

7 DFT calculation

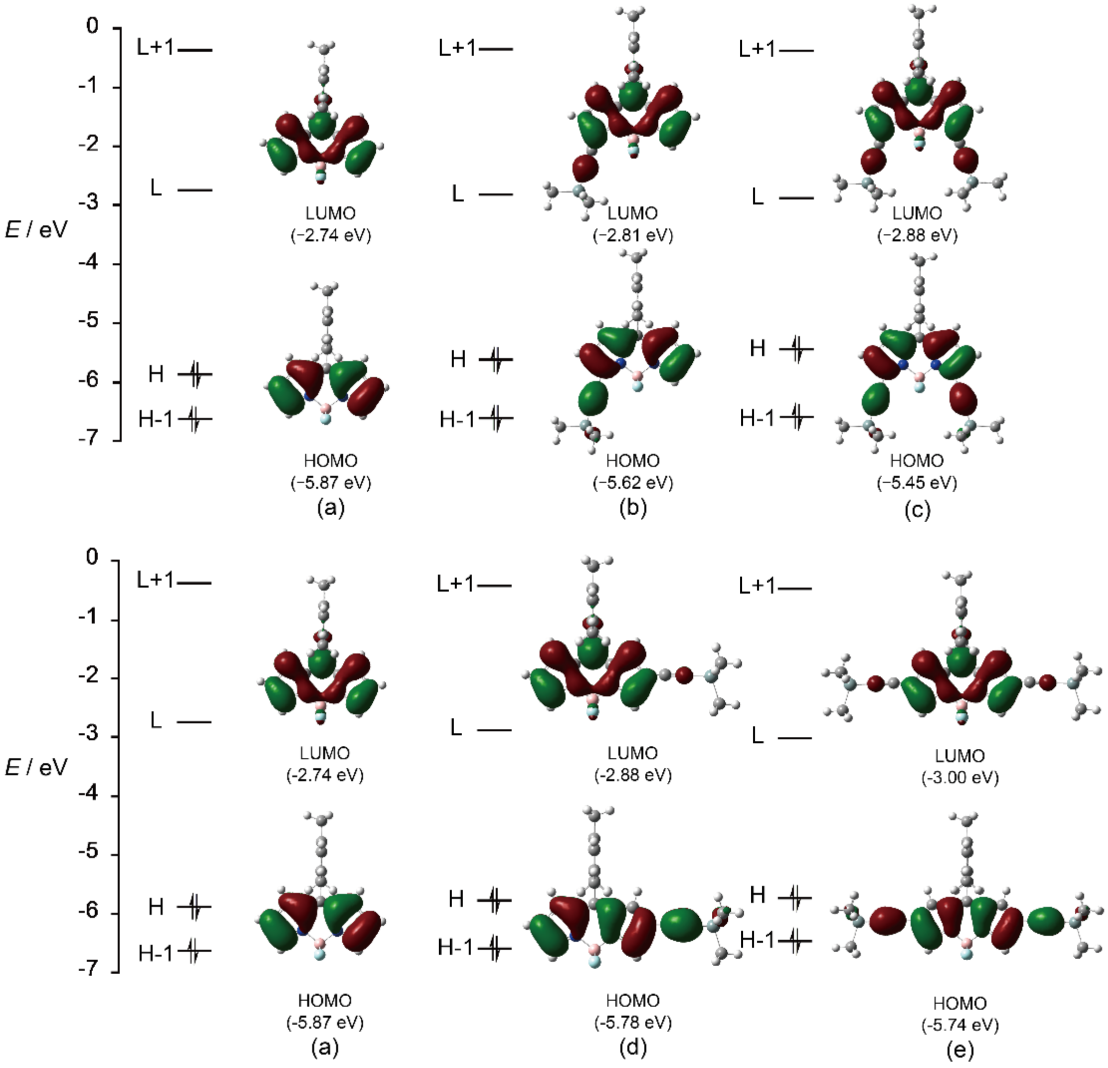

Figure S24: Energy diagrams and Kohn–Sham orbital representations of BODIPY derivatives, (a) **1a**, (b) **3a**, (c) **4a**, (d) **5a**, and (e) **6a**.

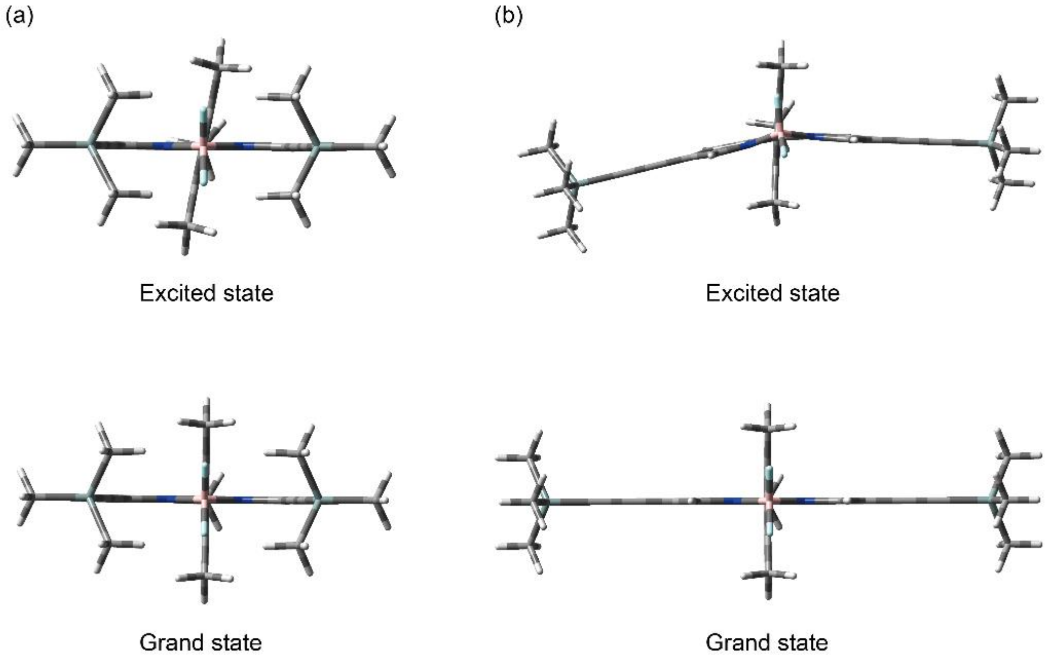

Figure S25: Excited-state (top) and grand-state (bottom) geometries of (a) **4a** and (b) **6a** obtained by time-dependent B3LYP/6-31G(d) level calculations.

References:

[S1] Lee, C. H.; S. Lindsey, J. *Tetrahedron* **1994**, *50*, 11427–11440. doi:10.1016/S0040-4020(01)89282-6

[S2] Brand, J. P.; Charpentier, J.; Waser, J. *Angew. Chem., Int. Ed.* **2009**, *48*, 9346–9349. doi:10.1002/anie.200905419

[S3] Nepomnyashchii, A. B.; Bröring, M.; Ahrens, J.; Bard, A. J. *J. Am. Chem. Soc.* **2011**, *133*, 8633–8645. doi:10.1021/ja2010219

[S4] a) Sheldrick, G. M. *Acta Crystallogr., Sect. A: Found. Adv.* **2015**, 71, 3–8. doi: 10.1107/S2053273314026370

b) Sheldrick, G. M. *Acta Crystallogr., Sect. C: Struct. Chem.* **2015**, 71, 3–8. doi: 10.1107/S2053229614024218

[S5] Gaussian 16, Revision A.03, M. J. Frisch, G. W. Trucks, H. B. Schlegel, G. E. Scuseria, M. A. Robb, J. R. Cheeseman, G. Scalmani, V. Barone, G. A. Petersson, H. Nakatsuji, X. Li, M. Caricato, A. V. Marenich, J. Bloino, B. G. Janesko, R. Gomperts, B. Mennucci, H. P. Hratchian, J. V. Ortiz, A. F. Izmaylov, J. L. Sonnenberg, D. Williams-Young, F. Ding, F. Lipparini, F. Egidi, J. Goings, B. Peng, A. Petrone, T. Henderson, D. Ranasinghe, V. G. Zakrzewski, J. Gao, N. Rega, G. Zheng, W. Liang, M. Hada, M. Ehara, K. Toyota, R. Fukuda, J. Hasegawa, M. Ishida, T. Nakajima, Y. Honda, O. Kitao, H. Nakai, T. Vreven, K. Throssell, J. A. Montgomery, Jr., J. E. Peralta, F. Ogliaro, M. J. Bearpark, J. J. Heyd, E. N. Brothers, K. N. Kudin, V. N. Staroverov, T. A. Keith, R. Kobayashi, J. Normand, K. Raghavachari, A. P. Rendell, J. C. Burant, S. S. Iyengar, J. Tomasi, M. Cossi, J. M. Millam, M. Klene, C. Adamo, R. Cammi, J. W. Ochterski, R. L. Martin, K. Morokuma, O. Farkas, J. B. Foresman, and D. J. Fox, Gaussian, Inc., Wallingford CT, **2016**.

[S6] a) A. D. Becke, *J. Chem. Phys.* **1993**, *98*, 5648–5652. doi:10.1063/1.464913

b) C. Lee, W. Yang, R. G. Parr, *Phys. Rev. B* **1988**, *37*, 785–789. doi:10.1103/PhysRevB.37.785

c) R. Ditchfield, W. J. Hehre, J. A. Pople, *J. Chem. Phys*. **1971**, *54*, 724–728. doi:10.1063/1.1674902

d) R. Ditchfield, *Mol. Phys.* **1974**, *27*, 789–807. doi:10.1080/00268977400100711

e) K. Wolinski, J. F. Hilton, P. Pulay, *J. Am. Chem. Soc*. **1990**, *112*, 8251–8260. doi:10.1021/ja00179a005

[S7] Hu, G.; Liu, R.; Alexy, E. J.; Mandal, A. K.; Bocian, D. F.; Holten, D.; Lindsey, J. S. *New J. Chem*. **2016**, *40*, 8032–8052. doi: 10.1039/c6nj01782g

Cartesian coordinates for **1a** (S_0)

SCF Done: E(RB3LYP) = -1030.38264725 A.U. after 1 cycles

Sum of electronic and thermal Enthalpies= -1030.045604

Sum of electronic and thermal Free Energies= -1030.115591

Stoichiometry C18H17BF2N2

Framework group C1[X(C18H17BF2N2)]

Deg. of freedom 114

Full point group C1 NOp 1

Largest Abelian subgroup C1 NOp 1

Largest concise Abelian subgroup C1 NOp 1

Standard orientation:

Center Number	Atomic Number	Atomic Type	Coordinates (Angstroms) X	Y	Z
1	6	0	2.557346	-2.528352	0.000050
2	6	0	1.430090	-3.377157	0.000181
3	6	0	0.312101	-2.553665	0.000198
4	6	0	0.781030	-1.213022	0.000123
5	7	0	2.175589	-1.243557	0.000020
6	1	0	3.607769	-2.788019	-0.000012
7	1	0	1.453487	-4.458188	0.000245
8	1	0	-0.728704	-2.847090	0.000291
9	6	0	0.078165	-0.000132	0.000130
10	6	0	0.778267	1.214359	0.000070
11	6	0	0.306285	2.553916	0.000097
12	7	0	2.172745	1.248077	-0.000018
13	6	0	1.422386	3.379974	0.000047
14	1	0	-0.735185	2.844928	0.000169
15	6	0	2.551570	2.533746	-0.000027
16	1	0	1.443321	4.461056	0.000062
17	1	0	3.601398	2.795803	-0.000088
18	6	0	-1.418461	-0.001546	0.000184
19	6	0	-2.117804	-0.004880	-1.224781
20	6	0	-2.117678	-0.004484	1.224901

21	6	0	-3.515836	-0.009834	-1.198347
22	6	0	-3.515978	-0.009418	1.198393
23	6	0	-4.234993	-0.008680	0.000143
24	1	0	-4.055781	-0.016071	-2.143135
25	1	0	-4.055956	-0.015264	2.143122
26	5	0	3.129595	0.003353	-0.000120
27	9	0	3.906287	0.004226	-1.143492
28	9	0	3.906580	0.004261	1.143050
29	6	0	-1.386992	-0.007374	-2.549219
30	1	0	-0.742205	0.872689	-2.655456
31	1	0	-0.741668	-0.887402	-2.652437
32	1	0	-2.094821	-0.008948	-3.383187
33	6	0	-1.387114	-0.006610	2.549479
34	1	0	-0.741822	-0.886619	2.653069
35	1	0	-0.742346	0.873478	2.655614
36	1	0	-2.095098	-0.007956	3.383315
37	6	0	-5.745680	0.017996	-0.000546
38	1	0	-6.123985	1.048527	-0.031681
39	1	0	-6.155471	-0.503683	-0.871954
40	1	0	-6.155278	-0.451048	0.900167

Cartesian coordinate for **3a** (S_0)

SCF Done: E(RB3LYP) = -1515.22746929 A.U. after 1 cycles

Sum of electronic and thermal Enthalpies= -1514.768252

Sum of electronic and thermal Free Energies= -1514.863148

Stoichiometry C23H25BF2N2Si

Framework group C1[X(C23H25BF2N2Si)]

Deg. of freedom 156

Full point group C1 NOp 1

Largest Abelian subgroup C1 NOp 1

Largest concise Abelian subgroup C1 NOp 1

Standard orientation:

Center Number	Atomic Number	Atomic Type	Coordinates (Angstroms) X	Y	Z
1	6	0	0.463237	3.558928	-0.000995
2	6	0	1.830592	3.911385	-0.001367
3	6	0	2.547755	2.723112	-0.000878
4	6	0	1.599383	1.664418	-0.000255
5	7	0	0.324109	2.226223	-0.000372
6	1	0	2.223138	4.918951	-0.001939
7	1	0	3.621526	2.595018	-0.000998
8	6	0	1.778395	0.275742	0.000102
9	6	0	0.660897	-0.573780	0.000237
10	6	0	0.602410	-1.994460	0.000228
11	7	0	-0.637353	-0.078710	0.000121
12	6	0	-0.732516	-2.348009	0.000067
13	1	0	1.462281	-2.650060	0.000253
14	6	0	-1.484798	-1.139020	0.000114
15	1	0	-1.162400	-3.339659	-0.000047
16	6	0	3.158050	-0.304030	0.000168
17	6	0	3.804609	-0.572283	-1.224619
18	6	0	3.804676	-0.571653	1.224775
19	6	0	5.095114	-1.109980	-1.198336
20	6	0	5.095391	-1.109457	1.198510
21	6	0	5.757552	-1.389577	0.000214
22	1	0	5.595253	-1.313876	-2.143031
23	1	0	5.595654	-1.312966	2.143184
24	5	0	-1.041512	1.445142	0.000750
25	9	0	-1.749295	1.752664	-1.143546
26	9	0	-1.747124	1.752152	1.146542
27	6	0	3.132269	-0.286124	-2.548999
28	1	0	2.196795	-0.847355	-2.656507
29	1	0	2.878136	0.775220	-2.651554
30	1	0	3.785555	-0.558201	-3.383160
31	6	0	3.132695	-0.284976	2.549226
32	1	0	2.878481	0.776385	2.651398

33	1	0	2.197314	-0.846261	2.657266
34	1	0	3.786250	-0.556617	3.383318
35	6	0	7.139583	-2.000139	-0.000347
36	1	0	7.088680	-3.096886	-0.027036
37	1	0	7.718376	-1.681737	-0.873899
38	1	0	7.700702	-1.723255	0.898334
39	6	0	-2.889693	-1.016442	0.000003
40	6	0	-4.109212	-0.950523	-0.000075
41	14	0	-5.942028	-0.734197	-0.000210
42	6	0	-6.419018	0.227136	1.553551
43	1	0	-6.142279	-0.319967	2.461707
44	1	0	-5.915443	1.199542	1.583507
45	1	0	-7.501363	0.404525	1.583971
46	6	0	-6.418844	0.226631	-1.554340
47	1	0	-5.915382	1.199090	-1.584483
48	1	0	-6.141874	-0.320701	-2.462288
49	1	0	-7.501205	0.403879	-1.585008
50	6	0	-6.750666	-2.442632	0.000014
51	1	0	-6.463690	-3.020779	0.885699
52	1	0	-7.844078	-2.352704	-0.000085
53	1	0	-6.463555	-3.021076	-0.885435
54	1	0	-0.407608	4.200990	-0.001173

Cartesian coordinates for **4a** (S_0)

SCF Done: E(RB3LYP) = -2000.07224932 A.U. after 1 cycles

Sum of electronic and thermal Enthalpies= -1999.491825

Sum of electronic and thermal Free Energies= -1999.609459

Stoichiometry C28H33BF2N2Si2

Framework group C1[X(C28H33BF2N2Si2)]

Deg. of freedom 198

Full point group C1 NOp 1

Largest Abelian subgroup C1 NOp 1

Largest concise Abelian subgroup C1 NOp 1

Standard orientation:

Center Number	Atomic Number	Atomic Type	Coordinates (Angstroms) X	Y	Z
1	6	0	0.048150	-2.555814	-0.000429
2	6	0	1.206059	-3.386203	-0.000205
3	6	0	2.304316	-2.550126	-0.000114
4	6	0	1.814005	-1.214290	-0.000237
5	7	0	0.426596	-1.252320	-0.000401
6	1	0	1.187223	-4.466893	-0.000118
7	1	0	3.349570	-2.826911	0.000069
8	6	0	2.517163	-0.001359	0.000044
9	6	0	1.815256	1.212301	0.000220
10	6	0	2.306925	2.547624	0.000857
11	7	0	0.427888	1.251760	0.000016
12	6	0	1.209539	3.384842	0.001046
13	1	0	3.352462	2.823295	0.001202
14	6	0	0.050783	2.555647	0.000398
15	1	0	1.191807	4.465552	0.001554
16	6	0	4.013747	-0.001865	0.000329
17	6	0	4.713935	-0.005046	1.225147
18	6	0	4.714342	-0.004057	-1.223962
19	6	0	6.112016	-0.009090	1.199334
20	6	0	6.112658	-0.008094	-1.197488
21	6	0	6.831339	-0.007205	0.000966
22	1	0	6.651898	-0.015127	2.144152
23	1	0	6.652970	-0.013294	-2.142025
24	5	0	-0.535996	0.000212	-0.001234
25	9	0	-1.301541	0.000424	1.144900
26	9	0	-1.298949	0.000803	-1.149116
27	6	0	3.983129	-0.008257	2.549484
28	1	0	3.337197	0.870944	2.656177
29	1	0	3.338384	-0.888701	2.653117
30	1	0	4.690737	-0.009177	3.383736
31	6	0	3.984319	-0.006258	-2.548734

32	1	0	3.339511	-0.886541	-2.653351
33	1	0	3.338580	0.873108	-2.655231
34	1	0	4.692418	-0.006719	-3.382570
35	6	0	8.341993	0.020446	0.001940
36	1	0	8.719869	1.051246	0.030841
37	1	0	8.752111	-0.499049	0.874538
38	1	0	8.752298	-0.450327	-0.897604
39	6	0	-1.292800	2.982838	0.000287
40	6	0	-1.295862	-2.981654	-0.000553
41	6	0	-2.446075	-3.392582	-0.000659
42	6	0	-2.442622	3.394855	0.000199
43	14	0	-4.220032	-3.899178	-0.000616
44	14	0	-4.216138	3.902981	0.000236
45	6	0	-4.309268	-5.787253	-0.000687
46	1	0	-5.353030	-6.125238	-0.000690
47	1	0	-3.821686	-6.210434	-0.886263
48	1	0	-3.821653	-6.210535	0.884820
49	6	0	-5.031068	-3.196062	-1.554441
50	1	0	-4.939720	-2.104824	-1.585161
51	1	0	-4.565207	-3.595011	-2.462434
52	1	0	-6.098414	-3.448535	-1.584696
53	6	0	-5.030918	-3.196138	1.553326
54	1	0	-4.564969	-3.595125	2.461257
55	1	0	-4.939548	-2.104901	1.584075
56	1	0	-6.098266	-3.448593	1.583682
57	6	0	-5.027702	3.200680	-1.553691
58	1	0	-4.561681	3.599616	-2.461609
59	1	0	-4.936968	2.109403	-1.584705
60	1	0	-6.094908	3.453769	-1.583802
61	6	0	-5.027693	3.200552	1.554107
62	1	0	-4.937201	2.109249	1.584910
63	1	0	-4.561504	3.599215	2.462060
64	1	0	-6.094839	3.453877	1.584358
65	6	0	-4.303825	5.791125	0.000318
66	1	0	-3.815950	6.214013	-0.885233
67	1	0	-5.347318	6.129943	0.000408

68	1	0	-3.815837	6.213922	0.885855

Cartesian coordinates for **5a** (S_0)

SCF Done: E(RB3LYP) = -1515.22885700 A.U. after 1 cycles

Sum of electronic and thermal Enthalpies= -1514.769403

Sum of electronic and thermal Free Energies= -1514.864773

Stoichiometry C23H25BF2N2Si

Framework group C1[X(C23H25BF2N2Si)]

Deg. of freedom 156

Full point group C1 NOp 1

Largest Abelian subgroup C1 NOp 1

Largest concise Abelian subgroup C1 NOp 1

Standard orientation:

Center Number	Atomic Number	Atomic Type	Coordinates (Angstroms) X	Y	Z
1	6	0	3.594295	-3.345398	-0.000219
2	6	0	4.729963	-2.506539	-0.000391
3	6	0	4.259975	-1.200964	-0.000325
4	6	0	2.840243	-1.267625	-0.000180
5	7	0	2.471976	-2.613734	-0.000098
6	1	0	4.837876	-0.287005	-0.000419
7	6	0	1.881396	-0.247257	-0.000134
8	6	0	0.514724	-0.569208	-0.000050
9	6	0	-0.626114	0.264668	-0.000066
10	7	0	0.083613	-1.898887	0.000036
11	6	0	-1.751798	-0.569056	-0.000016
12	1	0	-0.619677	1.345264	-0.000138
13	6	0	-1.250472	-1.900993	0.000061
14	6	0	2.309742	1.186273	-0.000168
15	6	0	2.511530	1.855554	1.225100

16	6	0	2.511197	1.855561	-1.225183
17	6	0	2.914250	3.194225	1.198502
18	6	0	2.913984	3.194476	-1.198481
19	6	0	3.117825	3.883791	-0.000095
20	1	0	3.073276	3.710301	2.143174
21	1	0	3.072717	3.710691	-2.143086
22	5	0	1.006261	-3.174523	0.000160
23	9	0	0.781596	-3.914393	1.143858
24	9	0	0.781347	-3.914802	-1.143222
25	6	0	2.304616	1.154682	2.549440
26	1	0	1.276813	0.788691	2.655967
27	1	0	2.963750	0.285034	2.653989
28	1	0	2.507656	1.832719	3.383398
29	6	0	2.304043	1.155039	-2.549672
30	1	0	2.963128	0.285394	-2.654558
31	1	0	1.276209	0.789126	-2.656148
32	1	0	2.506985	1.833280	-3.383488
33	6	0	3.521701	5.339600	0.000591
34	1	0	2.640957	5.994932	0.029897
35	1	0	4.136784	5.584803	0.872923
36	1	0	4.089271	5.598761	-0.899275
37	1	0	3.544312	-4.426287	-0.000193
38	1	0	-1.810638	-2.826193	0.000123
39	6	0	-3.118819	-0.199473	-0.000041
40	6	0	-4.299687	0.113084	-0.000059
41	14	0	-6.079818	0.579386	0.000006
42	6	0	-7.116194	-1.001191	-0.003154
43	1	0	-6.910201	-1.614953	0.881014
44	1	0	-8.187242	-0.763324	-0.003276
45	1	0	-6.909329	-1.611957	-0.889191
46	6	0	-6.445393	1.597479	1.550618
47	1	0	-6.231653	1.026940	2.461441
48	1	0	-5.840887	2.511163	1.579622
49	1	0	-7.501370	1.893931	1.580741
50	6	0	-6.444072	1.602804	-1.547408
51	1	0	-5.839290	2.516409	-1.572933

52	1	0	-6.229887	1.035274	-2.460005
53	1	0	-7.499944	1.899654	-1.577235
54	1	0	5.759304	-2.837513	-0.000533

Cartesian coordinates for **6a** (S_0)

SCF Done: E(RB3LYP) = -2000.07491948 A.U. after 1 cycles

Sum of electronic and thermal Enthalpies= -1999.493082

Sum of electronic and thermal Free Energies= -1999.613676

Stoichiometry C28H33BF2N2Si2

Framework group C1[X(C28H33BF2N2Si2)]

Deg. of freedom 198

Full point group C1 NOp 1

Largest Abelian subgroup C1 NOp 1

Largest concise Abelian subgroup C1 NOp 1

Standard orientation:

Center Number	Atomic Number	Atomic Type	Coordinates (Angstroms) X	Y	Z
1	6	0	-2.525012	-1.948929	0.000359
2	6	0	-3.387078	-0.815602	0.000407
3	6	0	-2.547570	0.304804	0.000230
4	6	0	-1.214775	-0.167430	0.000157
5	7	0	-1.247698	-1.565601	0.000207
6	1	0	-2.850034	1.342191	0.000214
7	6	0	-0.000094	0.533497	0.000029
8	6	0	1.214331	-0.167903	-0.000009
9	6	0	2.547283	0.303833	-0.000077
10	7	0	1.246742	-1.566076	0.000054
11	6	0	3.386396	-0.816874	-0.000058
12	1	0	2.850097	1.341104	-0.000121
13	6	0	2.523921	-1.949878	0.000016

14	6	0	0.000507	2.029407	-0.000085
15	6	0	-0.002008	2.727942	-1.225697
16	6	0	-0.001239	2.728095	1.225137
17	6	0	-0.004937	4.125798	-1.199039
18	6	0	-0.004155	4.126200	1.198102
19	6	0	-0.002582	4.844805	-0.000434
20	1	0	-0.010447	4.665727	-2.143698
21	1	0	-0.008996	4.666365	2.142589
22	5	0	-0.000661	-2.524804	0.000078
23	9	0	-0.000877	-3.295383	-1.143780
24	9	0	-0.000735	-3.295516	1.143844
25	6	0	-0.005620	1.997093	-2.549991
26	1	0	0.873796	1.351490	-2.657507
27	1	0	-0.887172	1.353926	-2.654597
28	1	0	-0.005959	2.704968	-3.383806
29	6	0	-0.004080	1.997772	2.549724
30	1	0	-0.885509	1.354551	2.655043
31	1	0	0.875464	1.352317	2.657064
32	1	0	-0.004079	2.705977	3.383258
33	6	0	0.026205	6.355280	-0.001287
34	1	0	1.057473	6.731604	-0.031135
35	1	0	-0.493655	6.765760	-0.873407
36	1	0	-0.443293	6.765774	0.898742
37	1	0	-2.797520	-2.995664	0.000441
38	1	0	2.796038	-2.996714	0.000045
39	6	0	4.801821	-0.855536	-0.000087
40	6	0	6.022669	-0.895254	-0.000111
41	6	0	-4.802516	-0.853774	0.000566
42	6	0	-6.023375	-0.893066	0.000623
43	14	0	7.862680	-0.956864	-0.000150
44	14	0	-7.863427	-0.953799	0.000511
45	6	0	-8.501855	-0.081367	1.551396
46	1	0	-8.181708	0.966369	1.580013
47	1	0	-9.598505	-0.097091	1.582159
48	1	0	-8.134282	-0.567723	2.461988
49	6	0	-8.501767	-0.076158	-1.547472

50	1	0	-8.134320	-0.559590	-2.459672
51	1	0	-9.598420	-0.091567	-1.578250
52	1	0	-8.181419	0.971604	-1.572657
53	6	0	-8.405652	-2.764147	-0.002524
54	1	0	-8.033217	-3.290639	-0.888526
55	1	0	-8.033433	-3.293537	0.881842
56	1	0	-9.500058	-2.841352	-0.002779
57	6	0	8.404033	-2.767468	0.002346
58	1	0	8.031442	-3.296439	-0.882114
59	1	0	9.498400	-2.845222	0.002454
60	1	0	8.031435	-3.294007	0.888252
61	6	0	8.501439	-0.084295	-1.550831
62	1	0	8.133579	-0.570208	-2.461543
63	1	0	8.181806	0.963608	-1.579135
64	1	0	9.598080	-0.100541	-1.581664
65	6	0	8.501600	-0.079939	1.548006
66	1	0	8.181846	0.967999	1.573465
67	1	0	8.133946	-0.563377	2.460118
68	1	0	9.598247	-0.095970	1.578706

Cartesian coordinates for **4a** (S_1)

SCF Done: E(RB3LYP) = -2000.07024673 A.U. after 1 cycles

Sum of electronic and thermal Enthalpies= -1999.401576

Sum of electronic and thermal Free Energies= -1999.523179

Stoichiometry C28H33BF2N2Si2

Framework group C1[X(C28H33BF2N2Si2)]

Deg. of freedom 198

Full point group C1 NOp 1

Largest Abelian subgroup C1 NOp 1

Largest concise Abelian subgroup C1 NOp 1

Standard orientation:

Center Number	Atomic Number	Atomic Type	Coordinates (Angstroms) X	Y	Z
1	6	0	-0.058537	-2.552780	0.047211
2	6	0	-1.218039	-3.389974	0.074860
3	6	0	-2.323547	-2.564590	0.061279
4	6	0	-1.841083	-1.217093	0.020342
5	7	0	-0.456465	-1.240990	0.013567
6	1	0	-1.192080	-4.470598	0.105457
7	1	0	-3.366911	-2.846254	0.084409
8	6	0	-2.569062	-0.001623	-0.000552
9	6	0	-1.842167	1.214437	-0.021385
10	6	0	-2.325860	2.561393	-0.064872
11	7	0	-0.457560	1.239667	-0.013413
12	6	0	-1.221134	3.387802	-0.078975
13	1	0	-3.369470	2.841973	-0.089590
14	6	0	-0.060848	2.551749	-0.048912
15	1	0	-1.196149	4.468389	-0.111653
16	6	0	-4.059280	-0.001873	-0.000435
17	6	0	-4.766230	-0.264921	-1.194955
18	6	0	-4.765624	0.256370	1.195597
19	6	0	-6.164269	-0.263226	-1.169341
20	6	0	-6.163594	0.247488	1.172347
21	6	0	-6.883286	-0.006288	0.001315
22	1	0	-6.704953	-0.464027	-2.092306
23	1	0	-6.703756	0.437499	2.097908
24	5	0	0.503270	-0.000175	0.004715
25	9	0	1.286070	-0.010989	-1.135805
26	9	0	1.274838	0.011391	1.152912
27	6	0	-4.037086	-0.531912	-2.492745
28	1	0	-3.335457	0.275506	-2.732958
29	1	0	-3.449000	-1.456620	-2.445780
30	1	0	-4.742767	-0.625491	-3.323711
31	6	0	-4.035522	0.517954	2.493947
32	1	0	-3.333835	-0.290367	2.730952
33	1	0	-3.447355	1.442739	2.449696

34	1	0	-4.740638	0.608938	3.325680
35	6	0	-8.393921	0.021560	-0.004121
36	1	0	-8.771918	1.030261	-0.218717
37	1	0	-8.804914	-0.647399	-0.767684
38	1	0	-8.804048	-0.278003	0.966238
39	6	0	1.278694	2.945528	-0.057291
40	6	0	1.281394	-2.945199	0.055748
41	6	0	2.461793	-3.285864	0.062513
42	6	0	2.458696	3.287556	-0.063897
43	14	0	4.274048	-3.607551	0.069757
44	14	0	4.270557	3.611434	-0.071587
45	6	0	4.573697	-5.473107	0.095662
46	1	0	5.648954	-5.691149	0.100251
47	1	0	4.135897	-5.936278	0.987126
48	1	0	4.138540	-5.960513	-0.784093
49	6	0	5.002904	-2.790707	1.611770
50	1	0	4.775597	-1.719221	1.629296
51	1	0	4.597060	-3.233902	2.527711
52	1	0	6.093752	-2.909678	1.632139
53	6	0	5.007806	-2.832458	-1.491260
54	1	0	4.605267	-3.300485	-2.396245
55	1	0	4.779929	-1.761985	-1.538538
56	1	0	6.098774	-2.951220	-1.504855
57	6	0	5.006811	2.830621	1.485354
58	1	0	4.604656	3.294363	2.392712
59	1	0	4.780148	1.759706	1.528389
60	1	0	6.097660	2.950514	1.498377
61	6	0	4.998945	2.802113	-1.617822
62	1	0	4.773340	1.730348	-1.639538
63	1	0	4.591431	3.248496	-2.531471
64	1	0	6.089583	2.922902	-1.638853
65	6	0	4.567897	5.477453	-0.090007
66	1	0	4.133192	5.960601	0.792316
67	1	0	5.642883	5.696821	-0.094943
68	1	0	4.128478	5.943856	-0.978986

Cartesian coordinates for **6a** (S_1)

SCF Done: E(RB3LYP) = -2000.06501180 A.U. after 1 cycles

Sum of electronic and thermal Enthalpies= -1999.407492

Sum of electronic and thermal Free Energies= -1999.530843

Stoichiometry C28H33BF2N2Si2

Framework group C1[X(C28H33BF2N2Si2)]

Deg. of freedom 198

Full point group C1 NOp 1

Largest Abelian subgroup C1 NOp 1

Largest concise Abelian subgroup C1 NOp 1

Standard orientation:

Center Number	Atomic Number	Atomic Type	Coordinates (Angstroms) X	Y	Z
1	6	0	-2.445916	-2.061673	-0.291010
2	6	0	-3.326185	-0.996082	-0.058678
3	6	0	-2.504591	0.222879	-0.071488
4	6	0	-1.214943	-0.172912	-0.309629
5	7	0	-1.201320	-1.569818	-0.450644
6	1	0	-2.858790	1.228965	0.100664
7	6	0	0.019034	0.606719	-0.304189
8	6	0	1.221571	-0.089335	-0.291771
9	6	0	2.557924	0.354554	-0.110662
10	7	0	1.252936	-1.501565	-0.398017
11	6	0	3.377965	-0.786711	-0.074960
12	1	0	2.870577	1.381635	0.007327
13	6	0	2.520989	-1.907177	-0.258653
14	6	0	-0.044961	2.091606	-0.216128
15	6	0	-0.272947	2.853515	-1.384610
16	6	0	0.106202	2.742313	1.028794
17	6	0	-0.339205	4.246247	-1.286434

18	6	0	0.032632	4.138568	1.078411
19	6	0	-0.184582	4.910130	-0.065901
20	1	0	-0.511762	4.827718	-2.190260
21	1	0	0.143095	4.634782	2.040848
22	5	0	0.064640	-2.401170	-0.842647
23	9	0	0.084244	-2.595442	-2.214783
24	9	0	0.076642	-3.601351	-0.157208
25	6	0	-0.426391	2.184010	-2.731795
26	1	0	0.449792	1.572620	-2.976637
27	1	0	-1.293182	1.513170	-2.755870
28	1	0	-0.553750	2.927150	-3.524928
29	6	0	0.331453	1.958198	2.302752
30	1	0	-0.421048	1.171701	2.430260
31	1	0	1.309064	1.461918	2.305851
32	1	0	0.286776	2.615569	3.176523
33	6	0	-0.226681	6.418748	0.008539
34	1	0	0.763474	6.853727	-0.183135
35	1	0	-0.912906	6.838637	-0.734971
36	1	0	-0.546003	6.762938	0.997969
37	1	0	-2.646976	-3.119700	-0.363572
38	1	0	2.784932	-2.953729	-0.317187
39	6	0	4.776858	-0.848798	0.102095
40	6	0	5.989709	-0.906392	0.255556
41	6	0	-4.700010	-1.041258	0.130432
42	6	0	-5.924539	-1.055361	0.301268
43	14	0	7.812692	-0.991793	0.484220
44	14	0	-7.756310	-1.078741	0.553183
45	6	0	-8.129321	-0.170623	2.167506
46	1	0	-7.774863	0.865363	2.134426
47	1	0	-9.211185	-0.150661	2.348545
48	1	0	-7.655053	-0.662752	3.023514
49	6	0	-8.545407	-0.182876	-0.912189
50	1	0	-8.314363	-0.682375	-1.859186
51	1	0	-9.636485	-0.164596	-0.798602
52	1	0	-8.196850	0.853185	-0.982412
53	6	0	-8.322824	-2.875550	0.635682

54	1	0	-8.082930	-3.412963	-0.288429
55	1	0	-7.846579	-3.407613	1.466621
56	1	0	-9.408565	-2.929962	0.782193
57	6	0	8.357770	-2.796007	0.343402
58	1	0	8.105636	-3.212904	-0.638172
59	1	0	9.443141	-2.885475	0.476610
60	1	0	7.874842	-3.418881	1.104801
61	6	0	8.644620	0.050362	-0.856474
62	1	0	8.399890	-0.320500	-1.858110
63	1	0	8.326763	1.097672	-0.801344
64	1	0	9.736035	0.024897	-0.746276
65	6	0	8.245153	-0.310387	2.194734
66	1	0	7.919990	0.730554	2.302623
67	1	0	7.766059	-0.894065	2.988804
68	1	0	9.329132	-0.342124	2.362043
